# Gamma-irradiated SARS-CoV-2 vaccine candidate, OZG-38.61.3, confers protection from SARS-CoV-2 challenge in human ACEII-transgenic mice

**DOI:** 10.1038/s41598-021-95086-4

**Published:** 2021-08-04

**Authors:** Raife Dilek Turan, Cihan Tastan, Derya Dilek Kancagi, Bulut Yurtsever, Gozde Sir Karakus, Samed Ozer, Selen Abanuz, Didem Cakirsoy, Gamze Tumentemur, Sevda Demir, Utku Seyis, Recai Kuzay, Muhammer Elek, Miyase Ezgi Kocaoglu, Gurcan Ertop, Serap Arbak, Merve Acikel Elmas, Cansu Hemsinlioglu, Ozden Hatirnaz Ng, Sezer Akyoney, Ilayda Sahin, Cavit Kerem Kayhan, Fatma Tokat, Gurler Akpinar, Murat Kasap, Ayse Sesin Kocagoz, Ugur Ozbek, Dilek Telci, Fikrettin Sahin, Koray Yalcin, Siret Ratip, Umit Ince, Ercument Ovali

**Affiliations:** 1 Acibadem Labcell Cellular Therapy Laboratory, Istanbul, Turkey; 2 Genetics and Bioengineering Department, Yeditepe University, Istanbul, Turkey; 3 Molecular Biology and Genetics Department, Uskudar University, Istanbul, Turkey; 4 Transgenic Cell Technologies and Epigenetic Application and Research Center (TRGENMER), Uskudar University, Istanbul, Turkey; 5 Animal Application and Research Center, Acibadem Mehmet Ali Aydinlar University, Istanbul, Turkey; 6 Medical Biochemistry Department, Acibadem Mehmet Ali Aydinlar University, Istanbul, Turkey; 7 Vocational School of Health Services, Acibadem Mehmet Ali Aydinlar University, Istanbul, Turkey; 8 Medical Biotechnology Department, Acibadem Mehmet Ali Aydinlar University, Istanbul, Turkey; 9 Histology and Embryology Department, Acibadem Mehmet Ali Aydinlar University, Istanbul, Turkey; 10 Medical Biology Department, Acibadem Mehmet Ali Aydinlar University, Istanbul, Turkey; 11 Biostatistics and Bioinformatics Department, Acibadem Mehmet Ali Aydinlar University, Istanbul, Turkey; 12 Medical Genetics Department, Acibadem Mehmet Ali Aydinlar University, Istanbul, Turkey; 13 Pathology Laboratory, Acibadem Maslak Hospital, Istanbul, Turkey; 14 Department of Pathology, School of Medicine, Acibadem Mehmet Ali Aydinlar University, Istanbul, Turkey; 15 Department of Medical Biology, Medical School of Kocaeli University, Kocaeli, Turkey; 16 Infectious Disease Unit, Acibadem Altunizade Hospital, Istanbul, Turkey; 17 Pediatric Bone Marrow Transplantation Unit, Medical Park Goztepe Hospital, Istanbul, Turkey; 18 Hematology Department, School of Medicine, Acibadem Mehmet Ali Aydinlar University, Istanbul, Turkey

**Keywords:** Infectious diseases, Pathogenesis

## Abstract

The SARS-CoV-2 virus caused the most severe pandemic around the world, and vaccine development for urgent use became a crucial issue. Inactivated virus formulated vaccines such as Hepatitis A and smallpox proved to be reliable approaches for immunization for prolonged periods. In this study, a gamma-irradiated inactivated virus vaccine does not require an extra purification process, unlike the chemically inactivated vaccines. Hence, the novelty of our vaccine candidate (OZG-38.61.3) is that it is a non-adjuvant added, gamma-irradiated, and intradermally applied inactive viral vaccine. Efficiency and safety dose (either 10^13^ or 10^14^ viral RNA copy per dose) of OZG-38.61.3 was initially determined in BALB/c mice. This was followed by testing the immunogenicity and protective efficacy of the vaccine. Human ACE2-encoding transgenic mice were immunized and then infected with the SARS-CoV-2 virus for the challenge test. This study shows that vaccinated mice have lowered SARS-CoV-2 viral RNA copy numbers both in oropharyngeal specimens and in the histological analysis of the lung tissues along with humoral and cellular immune responses, including the neutralizing antibodies similar to those shown in BALB/c mice without substantial toxicity. Subsequently, plans are being made for the commencement of Phase 1 clinical trial of the OZG-38.61.3 vaccine for the COVID-19 pandemic.

## Introduction

The development of a vaccine has the utmost biomedical priority due to the global COVID-19 pandemic caused by the SARS-CoV-2 virus. A safe and effective SARS-CoV-2 vaccine is urgently required to halt the global COVID-19 pandemic. Various COVID-19 vaccines have been widely used worldwide including Pfizer-BioNTech, Moderna, and other inactivated virus vaccines^1–4^. Small animal model systems are critical for better understanding the COVID-19 disease pathways and to determine medical precautions for improved global health, considering that there are currently no approved vaccines and only one antiviral approved for emergency use for SARS-CoV-2^5,6^. More significantly, several pioneering studies have shown that both SARS-CoV-2 and SARS-CoV use the same human angiotensin-converting enzyme 2 (hACE2) cellular receptor to enter cells^7–10^. The crystal structure of the SARS-CoV-2 S protein receptor-binding domain (RBD) which binds to hACE2 has been described, with an approximately 10- to 20-fold greater affinity toward hACE2 than SARS-CoV binds. Unfortunately, standard laboratory mice cannot be infected with SARS-CoV-2 due to the discrepancy of the S protein to the murine orthologous (mACE2) of the human receptor, making model development complicated^6,9^. Thus, wild-type C57BL/6 mice cannot be infected efficiently with SARS-CoV-2 because there is no hACE2 protein expressed that supports SARS-CoV-2 binding and infection. On the other hand, both young and aged hACE2 positive mice showed high viral loads in the lung, trachea, and brain upon intranasal infection in the literature^10–13^.

For understanding viral pathogenesis, vaccine production, and drug screening, animal models are crucial. To assess preclinical efficacy, non-human primates (NHPs) are the best animal models. The implementation of NHPs, however, is limited by the high costs, availability, and complexity of the necessary husbandry settings. For research and antiviral therapeutic progress, suitable small animal models are therefore important. Mouse models are popular because of their affordability, availability, and simple genetic structure, and have been commonly used to research human coronavirus pathogenesis^14,15^. As a cellular receptor, SARS-CoV-2 could use the ACE2 receptor of the human, bat, or civet but not the mouse^9,15^. Therefore, it seems that mice expressing hACE2 would be a conceivable choice for the vaccine challenge tests.

In this study, we tested our vaccine candidate (OZG-38.61.3) inactivated with gamma irradiation to assess their immunogenicity and protective efficacy against the SARS-CoV-2 viral challenge in K18-hACE2 mice and showed the efficacy of the vaccination in BALB/c mice. The vaccine candidate (OZG-38.61.3) was intradermally applied in the mice, which decreased the requirement of a higher amount of inactivated virus for proper immunization. K18-hACE2-transgenic mice, in which hACE2 expression is powered by the epithelial cell cytokeratin-18 (K18) promoter, were originally designed for the study of SARS-CoV pathogenesis and lead to a lethal infection model^13,16,17^. This study aimed to investigate whether the vaccinated transgenic mouse has a lower SARS-CoV-2 viral RNA copy number in nasal specimens along with increased humoral and cellular immune responses, including neutralizing antibodies to the virus, without experiencing substantial toxicity.

## Material and methods

### Human samples

In vitro isolation and propagation of SARS-CoV-2 from diagnosed COVID-19 patients were described in our previous study^18^. The study for SARS-CoV-2 genome sequencing was approved by the Ethics Committee of Acıbadem Mehmet Ali Aydınlar University (ATADEK-2020/05/41) and informed consent from the patients was obtained to publish identifying information/images. In addition, informed consent was obtained from the patients who participated in this study. These data do not contain any private information of the patients. All techniques had been executed according to the applicable guidelines.

### Manufacturing gamma-irradiated inactivated SARS-CoV-2 vaccine candidate

For the nasopharyngeal and oropharyngeal swab samples to have clinical significance, it is extremely important to comply with the rules regarding sample selection, taking into the appropriate transfer solution, transportation to the laboratory, and storage under appropriate conditions when necessary^18^. The production of a candidate vaccine for gamma-irradiated inactivated SARS-CoV-2 was reported in our previous report^19^. Isolation and propagation were performed from the samples taken on the 7th day when the viral load was predicted to be the most in patients diagnosed with COVID-19. During virus replication, 90% confluent Vero cells in cell culture flasks with a larger surface area were gradually cultured with virus-containing supernatant. The supernatants obtained at the end of the production were pooled and concentrated 10–15 times. To remove cellular wastes in the supernatant, diafiltration was performed. Finally, the concentrated virus was frozen before the 25 kGy gamma-irradiation processes. The inactivation status of the vaccine was confirmed by a 21-day Vero coculture MTT assay as reported in our previous study^19^. In this study, the last version of our vaccine candidate, OZG-38.61.3 was constituted from 10^13^ to 10^14^ viral copies of SARS-CoV-2 in a dose without adjuvant.

### Viral RNA extraction and viral genome sequencing

Viral RNA extractions were performed by Quick-RNA Viral Kit (Zymo Research, USA) in Acıbadem Labcell Cellular Therapy Laboratory BSL-3 Unit according to the manufacturer’s protocols. Library preparation was performed by CleanPlex SARS-CoV-2 Research and Surveillance NGS Panel (Paragon Genomics, USA) according to the manufacturer’s user guide. For the construction of the library, The CleanPlex^®^ Dual-Indexed PCR Primers for Illumina^®^ (Paragon Genomics, USA) were used by combining i5 and i7 primers. Samples were sequenced by Illumina MiSeq instrument with paired-end 131 bp long fragments. The data that passed the quality control were aligned to the reference genome (NC_045512.2) in Wuhan and a variant list was created with variant calling. The data analysis was described in detail in our previous study^20^.

### Nanosight

Nanoparticle Tracking Analysis (NTA) measurements were carried out for SARS-CoV-2 titer in suspension by using The NanoSight NS300 (Amesbury, UK). Samples were diluted with distilled water 1:10 ratio and transferred to Nanosight cuvette as 1 mL. Measurements were performed at room temperature with 5 different 60-s video recordings.

### Inactivated SARS-CoV-2 virus imaging by transmission electron microscopy

Viruses were inactivated and fixed with 2.5% glutaraldehyde in PBS (0.1 M, pH 7.2) for 2.5 h. One drop of glutaraldehyde-treated virus suspension was placed on the carbon-coated grid for 10 min. The remaining solution was absorbed with a filter paper and the grid was stained by a negative staining procedure. Then, it was evaluated under a transmission electron microscope (Thermo Fisher Scientific- Talos L120C) and photographed.

### In-solution tryptic digestion

In-solution digestion was performed according to the manufacturer's instructions using an ‘in-solution tryptic digestion and guanidination kit’ (#89895, Thermo Fisher Scientific, USA). The protocol can be summarized as follow: a 10 μg protein sample was added to 15 μL 50 mM Ambic containing 100 mM DTT solution. The volume was completed to 27 μL and incubated at 95 °C for 5 min. Iodoacetamide (IAA) was added to the heated sample to a 10 mM final concentration and incubated in the dark for 20 min. 1 μL of 100 ng/μL trypsin was then added and incubated for 3 h at 37 °C. 1 μL of 100 ng/μL trypsin was added to the peptide mixture and incubated overnight at 30 °C. After incubation, the solution was vacuum concentrated to dryness and the peptides were resuspended in 0.1% FA for the nLC-MS/MS analysis.

### Nano-liquid chromatography–mass spectrometry (Nlc-MS/MS) analysis

The peptides were analyzed by nLC-MS/MS using an Ultimate 3000 RSLC nanosystem (Dionex, Thermo Scientific, USA) coupled to a Q Exactive mass spectrometer (Thermo Scientific, USA). The entire system was controlled by Xcalibur 4.0 software (Thermo Fisher Scientific, USA). High-performance liquid chromatography(HPLC) separation was performed using mobiles phases of A (0.1% Formic Acid) and B (80% Acetonitril + 0.1% Formic Acid). Digested peptides were pre-concentrated and desalted on a trap column. Then the peptides were transferred to an Acclaim PepMap RSLC C18 analytical column (75 μm × 15 cm × 2 μm, 100 Å diameter, Thermo Scientific, USA). The gradient for separation was 6–32% B in 80 min, 32–50% B in 40 min, 50–90% B in 10 min, 90% in 15 min, 90–6% B in 10 min, and 6% B for 10 min with the flow rate of 300 nL/min. Full scan MS spectra were acquired with the following parameters: resolution 70.000, scan range 400–2000 m/z, target automatic gain control (AGC)3 × 106, maximum injection time 60 ms, spray voltage 2.3 kV. MS/MS analysis was performed by data-dependent acquisition selecting the top ten precursor ions. The instrument was calibrated using a standard positive calibrant (LTQ Velos ESI Positive Ion Calibration Solution 88323, Pierce, USA) before each analysis.

### LC–MS/MS data analysis

Raw data were analyzed with Proteom Discoverer 2.2 (Thermo Scientific, USA) software for protein identification and the following parameters were used; peptide mass tolerance 10 ppm, MS/MS mass tolerance 0.2 Da, mass accuracy 2 ppm, tolerant miscarriage 1, minimum peptide length 6 amino acids, fixed changes cysteine carbamidomethylation, unstable changes methionine oxidation, and asparagine deamination. The minimum number of peptides identified for each protein was considered to be 1 and obtained data were searched in the Uniprot/Swissprot database.

### Vero host cell protein ELISA

Residual Host Cell Protein (HCP) analysis in a viral product supernatant was performed with the manufacturer’s protocol of the Cygnustechnologies-VERO Cell HCP ELISA kit (F500). The absorbance was read at 450/650 nm with the microplate reader (Omega ELISA Reader).

### Vero DNA nanodrop

The vaccine candidate was solved in 100 cc pyrogen-free water. Firstly, pyrogen-free water was blanked and one drop sample was measured at the dsDNA program using Thermo Scientific NanoDrop™ One Spectrophotometers to determine Vero residual DNA and A260/A280 ratio for DNA/protein purity.

### Replicative competent coronavirus test with gamma-irradiated inactivated SARS-CoV-2 vaccine candidates

3 µg of lyophilized inactivated SARS-CoV-2 vaccine candidate in 100 µL pyrogen-free water was inoculated into %90 confluent Vero cells at 37 °C. The supernatant of this culture was replenished with fresh Vero cell culture every 3-to-5 days up to 21 days of incubation. As a negative control, only 100 µL pyrogen-free water was inoculated into Vero cells and cultured for 21 days with the same treatments. At the end of the incubation, the final supernatant was collected, centrifuged at 2000G for 10 min to remove cell debris. Next, the supernatants were concentrated 10 × with 100 kDa Amplicon tubes. The concentrated samples were tested in the xCelligence RTCA system in a dose-dependent manner as 10-1 to 10-6 to determine the cytopathic effect.

### SRID assay

5 μg/mL of SARS-COV-2 Spike S1 Monoclonal Antibody (ElabScience) antibodies were added to the gel at a concentration of 2%. Inactive SARS-CoV-2 was kept at room temperature for 15–30 min with 1% zwittergent detergent (mix 9 test antigens: 1 Zwittergent). Incubation was provided in a humid environment for 18 h. The gel was washed with PBS, taken on the glass surface, and covered with blotter paper, and kept at 37 °C until it dried. By staining the gel with Coomassie Brillant Blue, the presence of S antigen was determined according to the dark blue color (colorimetric).

### Quality control tests

Sterility was done in a BACTEC blood culture bottle along with the BACTEC™ FX blood culturing instrument (BD). The endotoxin level was determined with the Gel-clot endotoxin Limulus Amebocyte Lysate (LAL) test (Charles River Laboratories). Mycoplasma analysis was performed with the Mycoplasma species 500 PCR kit at GeneAmp PCR System 2700 (Applied Biosystems). Quality control tests of the vaccine including levels of chemistry analysis (Na, Cl, K, Ca) and Total Protein (The ADVIA 1800 Clinical Chemistry System, Siemens), osmolarity (Osmometer, freezing point depression), Ph, Glucose, Albumin (Dimension EX-L), sterility, mycoplasma, endotoxin level, and impurity assay were performed in Acıbadem Labmed Laboratory with accredited methods. Moisture Analyzer was performed at Yeditepe University with accredited methods.

### Quantitative RT-PCR to determine viral RNA copy number

Total RNA isolations were performed from SARS-CoV-2 specimens using Direct-zol RNA Miniprep Kits (Zymo Research, USA). Quantitative RT-PCR was performed with the QuantiVirus SARS-CoV-2 Test Kit (Diacarta) according to the manufacturer’s protocol. The quantitative RT-PCR analysis was analyzed in Roche Lightcycler 96.

### BALB/c mice test

For studies on BALB/c and B6.Cg-Tg(K18-hACE2)2Prlmn/J transgenic mice, we confirm that all methods were carried out following relevant guidelines and regulations. Furthermore, we confirmed that the study was carried out in compliance with the ARRIVE guidelines. To analyze the efficiency and toxicology of the dose of inactive vaccine candidate parallel to challenge, 15 Female BALB/c mice were utilized from AAALAC International accredited Acıbadem Mehmet Ali Aydinlar University Laboratory Animal Application and Research Center (ACUDEHAM; Istanbul, Turkey). All animal studies received ethical approval from the Acibadem Mehmet Ali Aydinlar University Animal Experiments Local Ethics Committee (ACU-HADYEK). BALB/c mice were randomly allocated into 3 groups, a negative control group (n = 5) and 2 different dose groups (dose of 1 × 10^13^ and 1 × 10^14^, n = 5 per group). To determine the immunogenicity with two different doses (dose 10^13^ and dose 10^14^, n = 5 per group) of inactive vaccine produced in Acibadem Labcell Cellular Therapy Laboratory, Istanbul, Turkey, on day 0 mice were vaccinated intradermally with the dose of 1 × 10^13^ and 1 × 10^14^ lyophilized vaccine candidate without adjuvant reconstituted in 100 cc pyrogen-free water and also control groups vaccinated with 100 cc pyrogen-free water. After 18 days booster dose was applied with the same vaccination strategies. Survival and weight change were evaluated daily and every week respectively. Blood samples were collected just before the sacrification on day 28 for serum preparation to be used for in vitro efficiency studies. Mice were sacrificed on day 28 post-immunization for analysis of B and T cell immune responses via SARS-Cov-2 specific IgG ELISA, IFNγ ELISPOT, and cytokine bead array analysis. Furthermore, dissected organs including the lungs, liver, kidneys of sacrificed mice were taken into 10% buffered formalin solution before they were got routine tissue processing for histopathological analysis. Also, the spleen tissues were taken into a normal saline solution including %2 Pen-Strep for T cell isolation following homogenization protocol.

### Transgenic mice for challenge test

5 female and 20 male B6.Cg-Tg(K18-hACE2)2Prlmn/J transgenic mice at 6 weeks of age were purchased from The Jackson laboratories. All animal experiments were approved by the Experimental Animal Committee of Acıbadem Mehmet Ali Aydınlar University (ACUHADYEK 2020/36). The mice housed in Transgenic Biosafety BSL-3 laboratories of AAALAC International accredited Acıbadem Mehmet Ali Aydinlar University Laboratory Animal Application and Research Center (ACUDEHAM; Istanbul, Turkey). Light, temperature, humidity, and feeding conditions followed the ACUDEHAM accredited operating procedures and also K18-hACE2 mice hospitalized in IVC systems (ZOONLAB BIO. A.S.) for 29-day challenge tests. Whole groups were identified as female and male in the base of the earring numbers start 40–64.

### Vaccination and challenge strategies

Transgenic mice were randomly allocated into 4 groups, negative control group (n = 5), positive control group (n = 6), and 2 different dose groups (dose of 1 × 10^13^ and 1 × 10^14^ viral particle, n = 7 per group). To determine the 21-day immunogenicity with two different doses (dose 10^13^ and dose 10^14^, n = 7 per group) of inactive vaccine produced in Acibadem Labcell Cellular Therapy Laboratory, Istanbul, Turkey, on day 0 mice were vaccinated intradermally with the dose of 1 × 10^13^ and 1 × 10^14^ SARS-CoV-2 viral RNA copy per microliter lyophilized vaccine without adjuvant reconstituted in 100 cc pyrogen-free water and both negative and positive control groups vaccinated with 100 cc pyrogen-free water. In whole groups, a booster dose of 1 × 10^13^ and 1 × 10^14^ SARS-CoV-2 viral RNA copy per microliter vaccine was administered intradermally on day 15 post-first vaccination. All animals were monitored daily for clinical symptoms, body-weight changes body temperature change (Supplementary Fig. 1 and Table 1). 25 days following vaccination, K18-hACE2 mice were intranasally infected with a 3 × 10^4^ TCID50 dose of infective SARS-CoV-2 in 30 µL solution in Biosafety level cabin II in Transgenic Animal Biosafety level 3 laboratory (ABSL-3) of AAALAC International accredited Acıbadem Mehmet Ali Aydinlar University Laboratory Animal Application and Research Center (ACUDEHAM; Istanbul, Turkey). TCID50 dose of SARS-CoV-2 was calculated in the previous study^19^. Starting from the day after the challenge, clinical symptoms, body-weight changes body temperature change controlled every 12 h. At 48 h after the challenge, the oropharyngeal swabs were collected from mice in all groups and analyzed for viral RNA copy number. After the mice were held in the postural position, the sample was taken by rotating the swab in the throat with a nasopharyngeal swap stick and then placed into the PCR analysis tube containing DNA/RNA shield solution, and then RT-PCR was performed to determine the SARS-CoV-2 RNA copy number. At 96 h after the challenge, the nasopharyngeal swabs and sera were collected from whole groups including negative control groups to analyze immunological and virological assays. Biopsy samples were collected including skin which was the vaccination part, brain, testis, ovarium, intestine, spleen, kidney, liver, lung, heart. Biopsy samples were collected and anatomically divided for qPCR analysis and histological and TEM examination.

### X-ray dark-field imaging of the lungs of SARS-CoV-2: infected K18-hACE2 mice

At 96 h after the challenge, whole mice of each group were imaged with the Siemens Arcadis Avantic C arms X-ray dark-field imaging system to evaluate the feasibility of early-stage imaging of acute lung inflammation in mice. All mice were anesthetized once with Matrx VIP 3000 Isoflurane Vaporizer (MIDMARK) system to obtain X-ray dark-field imaging of 3 mice from each group. All images were acquired as the posterior prone position of mice. The X-ray ran at 48 kV, distance to source grating 70 cm, 111°, and shooting with 0.2 and 0.3 mA. Following, the mice were euthanized for in vitro efficacy tests and histopathology analysis post-challenge on day 4. For serum collection, whole blood were isolated from facial vein and all mice were euthanized by exanguination and then, we decapitated after cervical dislocation.

### Histopathological applications

Transgenic mice and BALB/c mice were sacrificed on postimmunization for histopathology analysis. Dissected organs including the cerebellum, lungs, liver, kidneys, skin, intestine, and part of the spleen of sacrificed mice were taken into 10% buffered formalin solution before routine tissue processing for histopathological analysis after weighting. The histopathology analysis of the lung tissues of challenge mice groups was performed at the Department of Pathology at Acibadem Maslak Hospital.

### SARS-CoV-2 IgG ELISA

Before the sacrification, blood samples were collected from the whole group of mice. The serum was collected with centrifugation methods. Serum samples were stored at −40 C. To detect the SARS-COV-2 IgG antibody in mouse serum SARS-CoV-2 IgG ELISA Kit (Creative, DEIASL019) was used. Before starting the experiment with the whole sample, reagent and microplates pre-coated with whole SARS-CoV-2 lysate were brought to room temperature. As a positive control, 100 ng mouse SARS-CoV-2 Spike S1 monoclonal antibody was used (commercially available as E-AB-V1005, Elabscience). Serum samples were diluted at 1:64, 1:128, and 1:256 in a sample diluent, provided in the kit. Anti-mouse IgG conjugated with Horseradish peroxidase enzyme (mHRP enzyme) was used as a detector. After incubation with the stopping solution, the color change was read at 450 nm with the microplate reader (Omega ELISA Reader).

### Colorimetric microneutralization MTT assay

TCID50 (Median Tissue Culture Infectious Dose) of SARS-CoV-2 was determined by incubating the virus in a serial dilution manner with the Vero cell line (CCL81, ATCC) in gold microelectrodes embedded microtiter wells in xCELLigence Real-Time Cell Analysis (RTCA) instruments (ACEA, Roche) for 8 days^19^. Neutralization assay of sera from transgenic and BALB/c mice groups was performed at 1:128, and 1:256 dilutions pre-incubated with a 100X TCID50 dose of SARS-CoV-2 at room temperature for 60 min. Because the sensitivity and specificity of the colorimetric microneutralization assay were shown closely related to the gold standard tests with plaque reduction neutralization test (PRNT), we wanted to quantify the neutralization capacity of the immunized mice serum on ELISA. Next, the pre-incubated mixture was inoculated into the Vero-cell-coated flat-bottom 96-well plate which was analyzed at the end of 96 h following standard MTT protocol. Viable cell analysis was determined by colorimetric change at the ELISA system. The neutralization ratio was determined by assessing percent neutralization by dividing the value of serum-virus treated condition wells by the value of untreated control Vero cells. 100% of neutralization was normalized to only the Vero condition while 0% of neutralization was normalized to the value of only 100 × TCID50 dose of SARS-CoV-2 inoculated Vero cell condition. For example, for the sample of 1:128 serum sample, the value was 0.651 while the value for control Vero well was 0.715, and the value for control SARS-CoV-2 inoculated well was 0.2. The calculation is as % neutralization = ((0.651 − 0.2)*100)/(0.715 − 0.2). This gave 87.5% virus neutralization. This calculation was performed for each mouse in the group and the mean of the virus neutralization was determined.

### Mouse IFN-γ ELISPOT analysis

Mouse Spleen T cells were centrifuged with Phosphate Buffer Saline (PBS) at 300 × g for 10 min. Pellet was resuspended in TexMACs (Miltenyi Biotech, GmbH, Bergisch Gladbach, Germany) cell culture media (%3 human AB serum and 1% Pen/Strep). 500,000 cells in 100 µL were added into a microplate already coated with a monoclonal antibody specific for mouse IFN-γ. 1000 nM SARS-CoV-2 virus Peptivator pool (SARS-CoV-2 S, N, and M protein peptide pool) (Miltenyi Biotech, GmbH, Bergisch Gladbach, Germany) were added into each well including mouse spleen T cells. The microplate was incubated in a humidified 37 °C CO_2_ incubator. After 48 h incubation, IFN-γ secreting cells were determined with Mouse IFNγ ELISpot Kit (RnDSystems, USA) according to the manufacturer’s instructions. The spots were counted under the dissection microscope (Zeiss, Germany).

### Unstimulated/stimulated T cell cytokine response and immunophenotype

500,000 cells isolated from mouse spleen were incubated with 1000 nM SARS-CoV-2 virus Peptivator pool (SARS-CoV-2 S, N, and M protein peptide pool) (Miltenyi Biotech, GmbH, Bergisch Gladbach, Germany) in a humidified 37 °C CO_2_ incubator. After 48 h incubation, the mouse cytokine profile was analyzed using the supernatant of the cultures using the MACSPlex Cytokine 10 kit (Miltenyi Biotec). Also, to determine T cell activation and proliferation, the restimulated cells were stained with the antibodies including CD3, CD4, CD8, and CD25 as an activation marker (Miltenyi Biotec). The Cytokine bead array and the T cell activation and proportions were analyzed using the MACSQuant Analyzer (Miltenyi Biotec).

### Statistics

Normally distributed data in bar graphs was tested using student’s t-tests for two independent means. The Mann–Whitney U test was employed for comparison between two groups of non-normally distributed data. Statistical analyses were performed using the Graphpad Prism and SPSS Statistics software. Each data point represents an independent measurement. Bar plots report the mean and standard deviation of the mean. The threshold of significance for all tests was set at *p < 0.05. *ns* is non-significant.

## Results

### Characterization of inactivated SARS-CoV-2 virus constituting OZG-38.61.3 vaccine candidates

**Figure 1:**
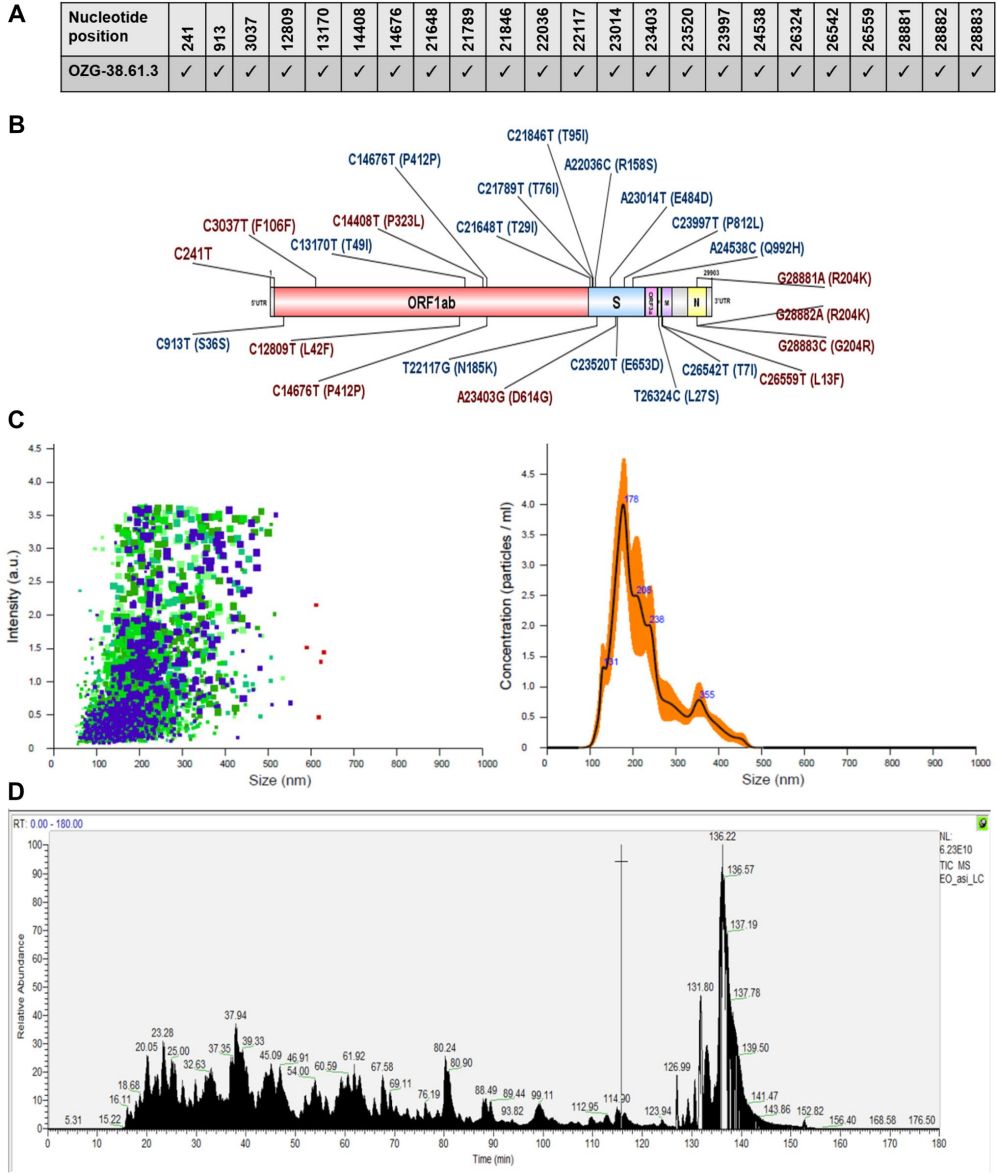

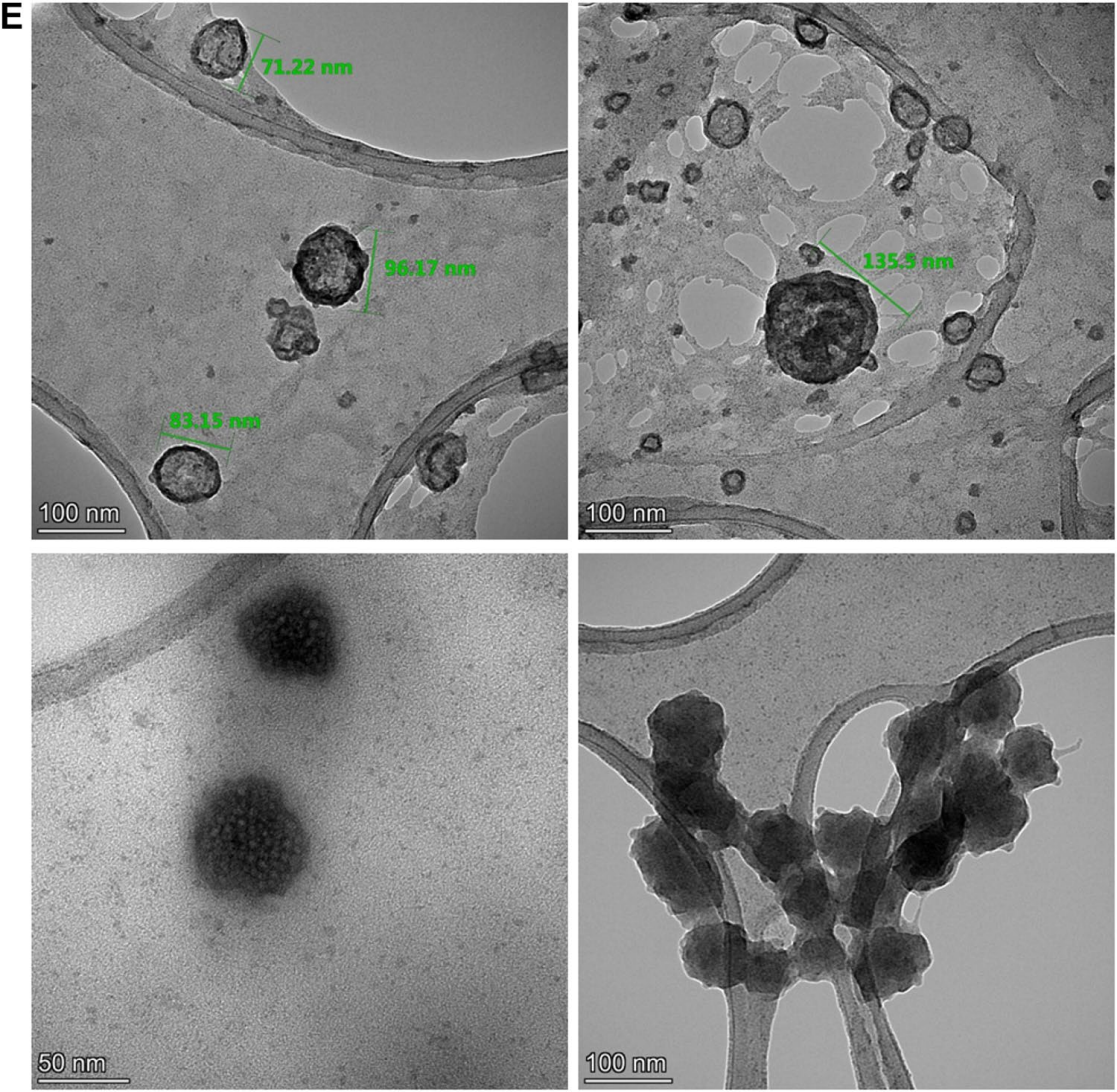
Characterization of inactivated SARS-CoV-2 virus constituting OZG-38.61.3 vaccine candidates. (**A**) Mutation distribution of SARS-CoV-2 virus strain that makes up the OZG-38.61.3 vaccine candidate. (**B**) Representation of variants detected in the virus strain that makes up the OZG-38.61.3 vaccine candidate on the SARS-CoV-2 genome. (**C**) The left plot showing intensity versus the size of the particles in OZG-38.61.3. The right plot showing the means of particle size of the candidate in the sample read three times. (**D**) Proteome analysis of inactivated OZG-38.61.3 SARS-CoV-2 product. (**E**) TEM image of SARS-CoV-2 Virus. Representative electron micrographs of SARS-CoV-2. Virus particles were seen on the grid (Scale bars: 50 nm, 100 nm).

One of the conclusions of our previous study was that the adjuvant positive vaccine administration should be removed from the newly designed version of the OZG-38.61 vaccine model as it caused an inflammatory reaction in the skin, cerebellum, and kidney in toxicity analysis of vaccinated mice^19^. Hence, it was decided to increase the SARS-CoV-2 effective viral RNA copy dose (1 × 10^13^ or 1 × 10^14^ viral copies per dose) without an adjuvant in this new version of the OZG-38.61 vaccine. Firstly, we determined whether this vaccine product comprises all identified SARS-CoV-2 mutations. The obtained sequences from the propagated SARS-CoV-2 virus were compared with the GISAID database and the protein levels of the variant information were examined (Fig. 1A). We determined that the SARS-CoV-2 strain forming OZG-38.61.3 vaccine covered previously identified mutation variants (red-colored) with new variants (blue-colored) (Fig. 1B). The data of all defined mutations were presented in detail in Supplementary Table 2. Using Nanosight technology, we determined that the size of inactivated SARS-CoV-2 was 187.9 ± 10.0 nm (mode) with a concentration of 4.23 × 10^9^ ± 1.88 × 10^8^ particles/mL (Fig. 1C). As a result of the LC–MS–MS analysis, the presence of proteins belonging to the SARS-CoV-2 virus was detected in the analyzed sample (Fig. 1D). Quality control tests are illustrated in Supplementary Table 3. Four of the defined proteins were Master Proteins and have been identified with high reliability by LC–MS/MS (Supplementary Table 4). Other proteins were Master Candidate proteins and their identification confidence interval is medium. The data of all defined proteins were presented in detail in Supplementary Table 4. Also, the transmission electron microscope was evaluated with the negative staining method and the main structures (envelope/spike) of the SARS-CoV-2 virus particles in the final product were well preserved (Fig. 1E). Also, TEM analysis confirmed the virus size was 70–200 nm with the presence of aggregates as determined in the Nanosight analysis. On the other hand, the concentration of the Vero host cell protein per vaccine dose was determined < 4 ng, and Vero host DNA per dose was undetectable. According to all these analyses, OZG-38.61.3 has been shown to pass all vaccine development criteria in the final product (Supplementary Table 3), comprise all the mutations identified up to date, preserve the protein structure and contain pure inactive virus free of residues.

### Acute toxicity and efficacy study of OZG-38.61.3 in BALB/C mice

**Figure 2:**
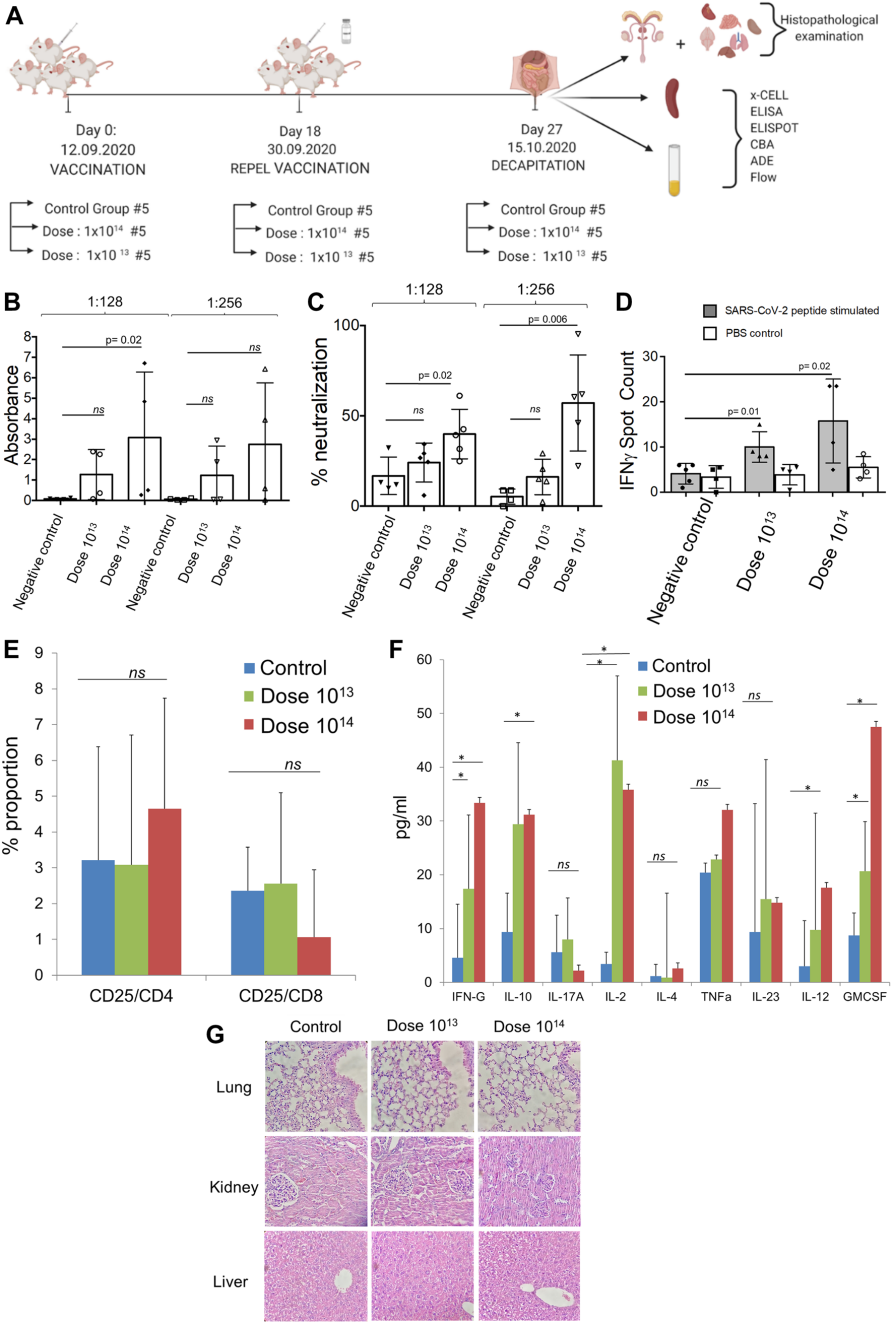
In vivo efficacy analysis of dose 10^13^ and dose 10^14^ in vaccinated BALB/c mice. (**A**) Representation of in vivo experimental setup of the BALB/c mice vaccinated with dose 10^13^ or dose 10^14^ of OZG-38.61.3. (**B**) The bar graph showing the presence (absorbance) of SARS-CoV-2 specific IgG in the mice sera diluted with either 1:128 or 1:256 detected using ELISA. (**C**) The bar graph showing the neutralization frequency of the mice sera diluted to 1:128 or 1:256 that were pre-incubated with a 100 × TCID50 dose of infective SARS-CoV-2. The analysis was performed with MTT analysis at 96 h. (**D**) The bar graph showing activation frequency (IFNγ positive spot count) of spleen T cells that were incubated with SARS-CoV-2 specific peptides. (**E**) The bar graph showing the proportion of the activated T cells after the stimulation. The activation was determined with the upregulation of the CD25 surface marker. (**F**) The bar graph showing cytokine proportions of spleen T cells that were incubated with SARS-CoV-2 specific peptides. (**G**) Histopathologic analysis of the lung, kidney, and liver tissues of BALB/c mice groups. H&E stain X400.

Acute toxicity and efficacy assays were studied in BALB/c mice. There were 3 groups in this study: control group (n = 5), dose 10^13^ (n = 5), and dose 10^14^ (n = 5) (Fig. 2A). There was no difference in nutrition and water consumption between the groups, as well as in total body weight and organ weight (Supplementary Fig. 1). Version 3 of the OZG-38.61 vaccine did not differ in the histopathologic analysis from the control group, including the dose 10^14^ group (Supplementary Table 5). Firstly, SARS-CoV-2 specific IgG at 1:128 dilution of serum isolated from mice groups showed a significant increase in the dose 10^14^ group in comparison with the control group (Fig. 2B). Since the standard deviations of the data in these studies were large and the spontaneous neutralizing activity in neutralizing antibody analyzes reached 80% in the study, dose 10^15^ and dose 10^16^ were studied with rodents of 10 females and 10 males in each group to test the accuracy of the data. Serum samples obtained on day 21 were used in the Repeated Dose study design in rodents. Serum samples were diluted in 1:32, 1:64, 1:28, 1:256 dilution factors and serums were studied with the Creative Diagnostic SARS-CoV-2 Specific IgG Elisa kit. It has also been shown in rodents that SARS-CoV-2 IgG antibodies can be formed at high rates (Supplementary Fig. 2). Secondly, to determine the neutralization capacity of serum collected from the immunized mice, 1:128 and 1:256 dilutions of sera were pre-inoculated with the SARS-CoV-2 virus, and following 96-h incubation, MTT analysis was performed. Findings showed that the dose 10^14^ vaccinated mice managed to neutralize the virus at a statistically significant level (*p* < 0.05) at both dilutions (Fig. 2C). Furthermore, we also determined the neutralization capacity of the OZG-38.61.3 vaccine administrated rodents treated with placebo, dose 1 × 10^13^, dose 1 × 10^14^ vaccine by diluting 1:32 to 1:256 (Supplementary Fig. 3), which suggested that repeated doses of the vaccine candidate were developing adequate immune response against the SARS-CoV-2. The BALB/c study showed that upon restimulation, gamma interferon secretion of the T lymphocytes from both vaccination groups increased significantly in comparison with the non-vaccinated mice group and PBS non-stimulated internal control groups (Fig. 2D). Furthermore, spleen T cells stimulated with the peptides were analyzed through flow cytometry to determine proportions of activated (CD25+) CD4+ and CD8+ T cells, but no proliferation was observed (Fig. 2E). Next, the supernatant of the incubated cells was analyzed using a cytokine bead array for a more detailed examination. Both doses of OZG-38.61.3 (especially dose 10^14^) increased IL-2, GM-CSF, gamma-IFN levels and caused Th-1 response (Fig. 2F). At the same time, IL-10 was increased in both dose groups, suggesting that the Tr1 (Regulatory T lymphocyte type 1 response) response was stimulated (Fig. 2F)^21^. Moreover, we performed histopathology analysis of the lung, liver, and kidney to determine inflammation, hemorrhage, and eosinophil infiltration (Fig. 2G and Supplementary Table 5). There was no significant toxicity including hemorrhage and eosinophil infiltration in overall organs (Fig. 2G and Supplementary Table 5). Therefore, we could not find any significant difference in the induction of SARS-CoV-2 specific antibodies and neutralizing antibody levels in the 1 × 10^13^ doses of the OZG-38.61.3 immunization group versus the negative control group (Fig. 2B, C). Besides, Dose 10^14^ of OZG-38.61.3 led to the effective neutralizing SARS-CoV-2 specific IgG antibody production and cytokine secretion, hence a satisfactory Th1 response, without significant toxicity.

### Challenge test with OZG-38.61.3 vaccinated humanized ACEII+ mice

**Figure 3:**
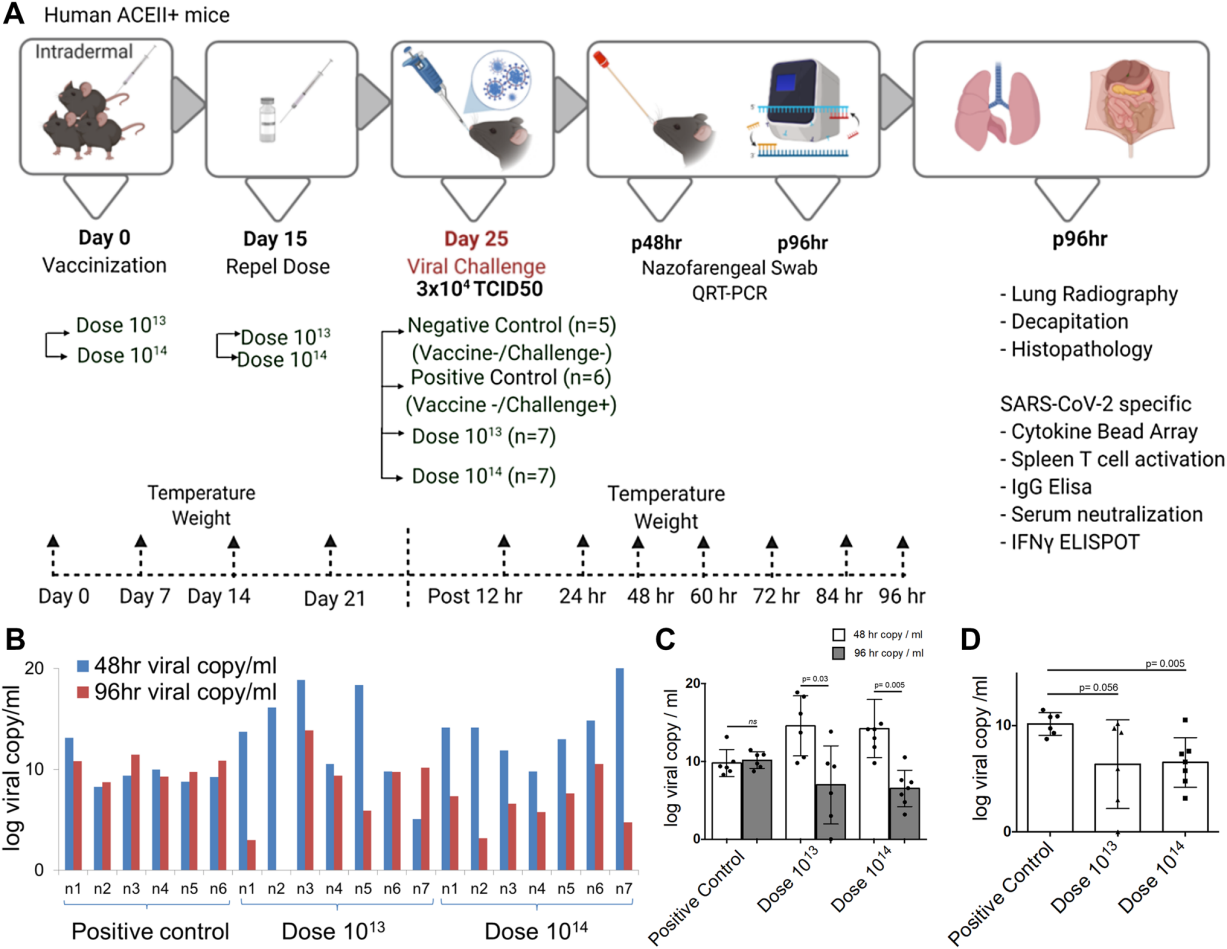
Challenge test with OZG-38.61.3 vaccinated humanized ACEII+ mice. (**A**) Representation of in vivo experimental setup of the challenge test. (**A**) Mice were allocated into 4 groups, a negative control group (n = 5), a positive control group (n = 6), and 2 different intradermally vaccinated groups (dose 10^13^ and dose 10^14^, n = 7 per group). A booster dose of dose 10^13^ and dose 10^14^ vaccine was administered on day 15. After 25 days of vaccination, the mice were intranasally infected with a 3 × 10^4^ TCID50 dose of SARS-CoV-2. Biopsy samples, spleen T cells, and serum were collected after euthanization at 96 h. (**B**) The bar graph showing SARS-CoV-2 viral RNA copy number in log scale per ml of the nasopharyngeal samples collected from each mouse at 48 h and 96 h post-challenge that were either vaccinated with dose 10^13^ (n = 7), dose 10^14^ (n = 7), or without vaccination group (positive control; n = 6). (**C**) The bar graph shows the mean value of viral RNA copy in log scale per ml at 48 h and 96 h post-challenge. (**D**) The bar graph showing a comparison of viral RNA copy number at 96 h between vaccinated and positive control groups.

Following efficacy and safety analysis of OZG-38.61.3 in BALB/c mice, post-immunization protection from SARS-CoV-2 infection in human ACE2 expressing transgenic mice was determined. Viral challenge analyzes were performed in K18-hACE2 (Jackson Lab). Two mice groups were vaccinated with the 10^13^ and 10^14^ doses of the vaccine. Mice were euthanized for in vitro efficacy tests and histopathology analysis post-challenge on day 4 (Fig. 3A). During the challenge, there was no significant change in food and water consumption along with temperature (Supplementary Fig. 1). Also, although not statistically significant, weight distribution was more uniform in vaccine groups (Supplementary Fig. 1 and Table 1). We recorded weight every 4-to-5 days till day9 and continued as day 14 and day 21. Before characterization tests, we recorded the weight per each 12 h as Day 26, 27, 28, and 29. Thus, we showed that there is not any weight change due to immunizations.

**Figure 4:**
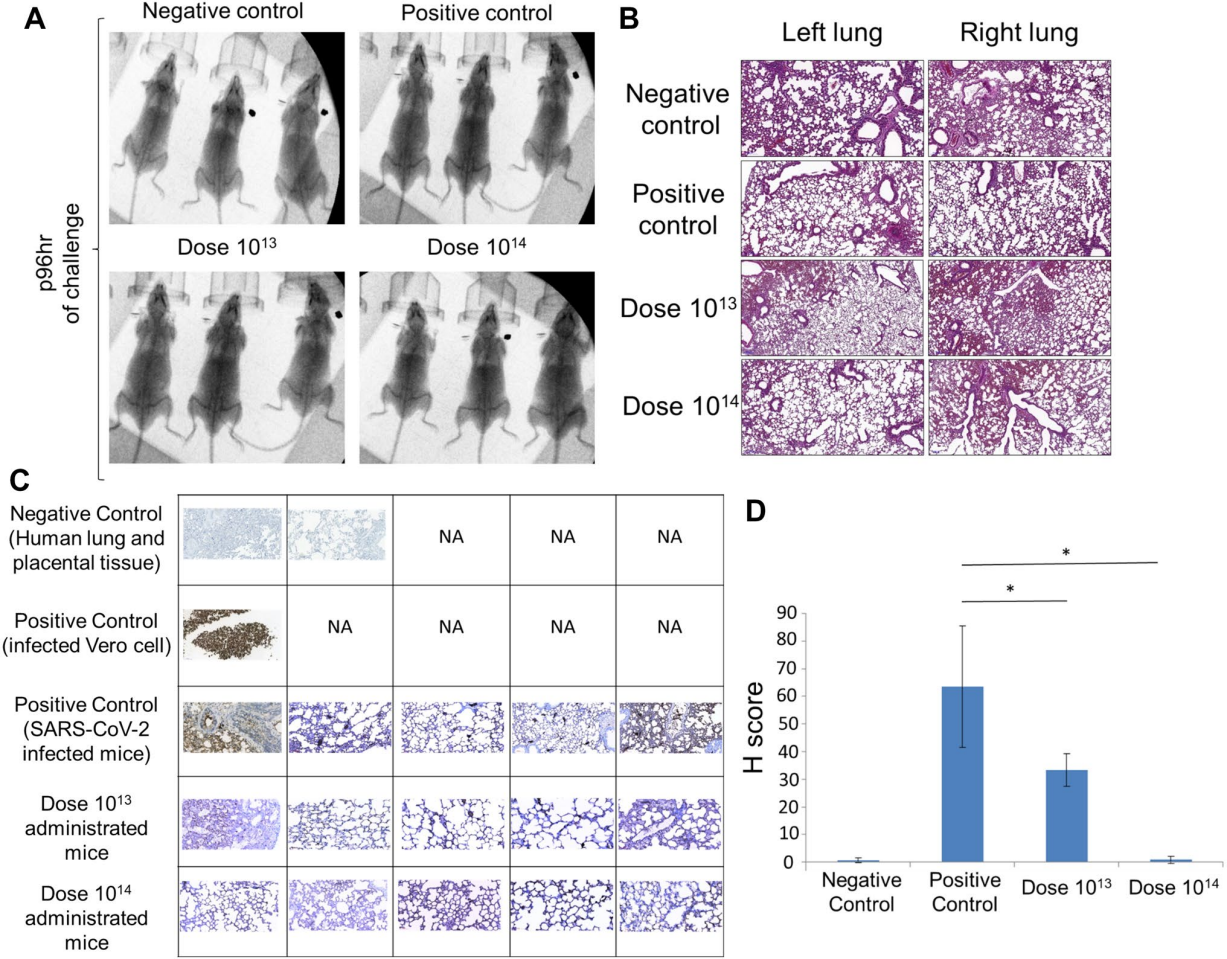
X-ray imaging and histopathology analysis of the lungs of SARS-CoV-2 infected mice. (**A**) X-ray imaging and (**B**). Histopathology analysis of the mice groups that were negative control (uninfected and no vaccination), positive control (only infection), dose 10^13^ group, and dose 10^14^ group (vaccinated and infected). (**C**) Immunohistochemistry analysis of the lung tissues. Paraffin block was prepared from SARS-CoV-2 cell culture and lung tissues of the challenge assay mice groups. Sections of 4 microns made from paraffin block were studied immunohistochemically with SARS/SARS-CoV-2 coronavirus nucleocapsid monoclonal antibody (B46F) (ThermoFisher Scientific, US). Infected Vero cells were used as positive control; Human lung and placental tissue were used as negative controls (50 µm). *NA* not applied. (**D**) Immunohistochemistry analysis (H-score) of the lung tissues. *p < 0.05.

This was followed by viral load analyzes on oropharyngeal swab samples on the 2nd and 4th days of the challenge test. While there was no significant change in the virus load in the positive control group, it was observed that the virus load decreased in the vaccine groups except for only one mouse, whereas it completely disappeared in one (Fig. 3B). It was observed that the mean virus load decreased statistically significantly over time, especially in the dose 10^14^, between 48 and 96 h. There was no change in the positive control group (Fig. 3C). When compared with the positive control group at the 96th hour, a 3-log decrease in viral RNA copy number was determined especially in the dose 10^14^ vaccine group (Fig. 3D). No difference was observed between the groups in the lung X-ray imaging analysis of the mice groups taken in our study (Fig. 4A). Also, it is observed that both vaccine doses do not cause antibody-dependent enhancement (ADE) side effects in the lung in histopathology analysis (Fig. 4B). No histologically significant change was observed in the positive control and vaccine groups, although positive control has signs of partial alveolar fusion and inflammation in 1 mouse (Fig. 4B). This finding is similar to chest radiographs. The absence of an additional pathology in the lung, especially in vaccine groups, was another additional finding confirming that ADE does not occur and the inactivity of our vaccine. Statistically significant reduction of viral load in the lung tissues correlated with increasing doses of the vaccine was determined by immunohistochemistry analysis (Fig. 4C) with histological scoring (H-score) (Fig. 4D and Supplementary Table 6). Thus, viral load analyses in the oropharyngeal specimens along with the histological analysis of the lung tissues showed that the SARS-CoV-2 infection was significantly reduced in the vaccinated groups.

### In vitro efficacy analysis of serum and T cells isolated from mice following challenge test

**Figure 5:**
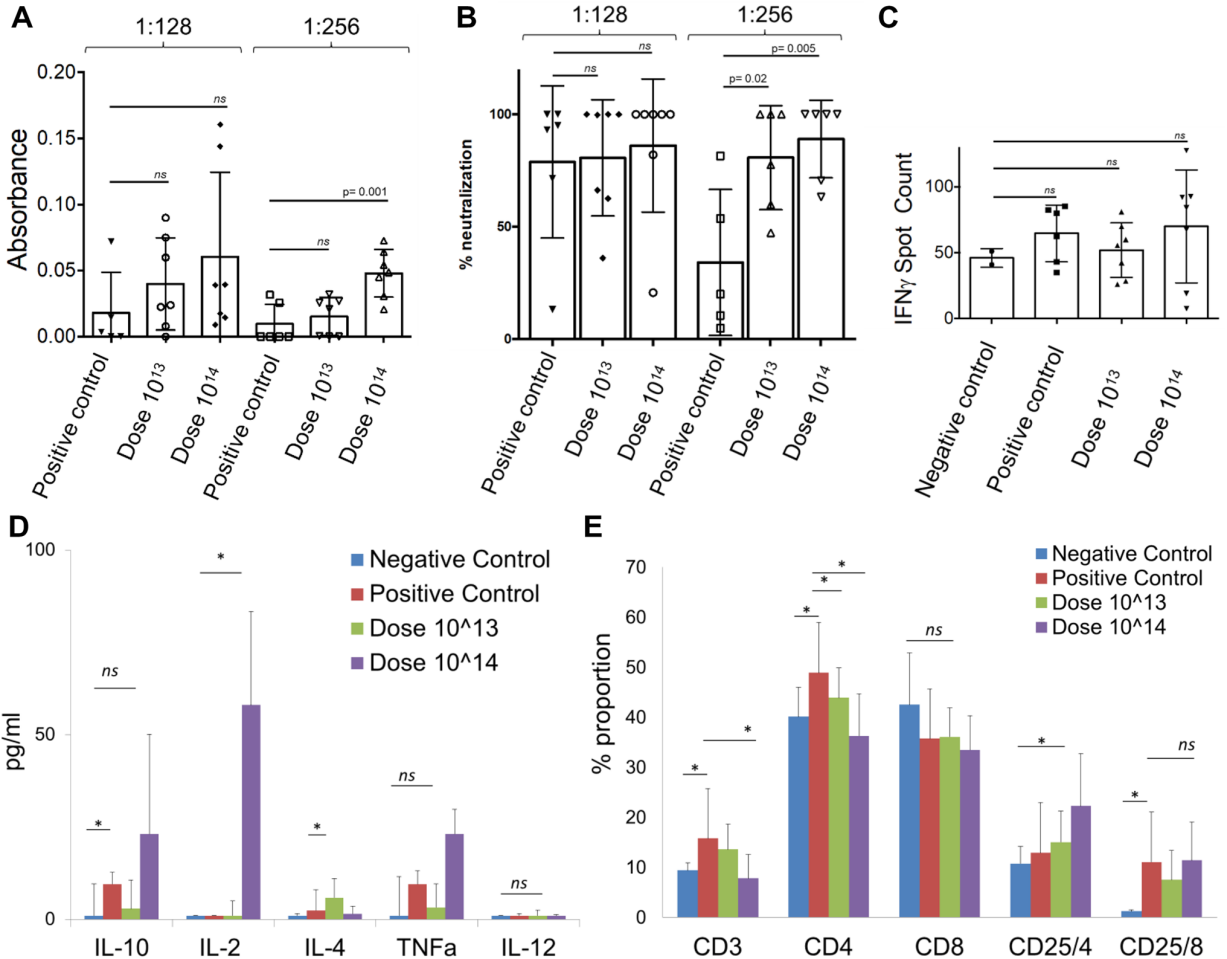
In vitro efficacy analysis of serum and spleen T cell isolated from challenge applied mice. (**A**) The bar graph showing the presence (absorbance) of SARS-CoV-2 specific IgG in the mice sera diluted with either 1:128 or 1:256 detected using ELISA. (**B**) The bar graph showing the neutralization frequency of the mice sera diluted to 1:128 or 1:256 that were pre-incubated with a 100 × TCID50 dose of infective SARS-CoV-2. The analysis was performed with MTT analysis at 96 h. (**C**) The bar graph showing the activation frequency (IFNγ positive spot count) of spleen T cells that were incubated with SARS-CoV-2 specific peptides. (**D**) The bar graph showing cytokine (IL-10, IL-2, IL-4, TNFa, and IL-12) proportions of spleen T cells that were incubated with SARS-CoV-2 specific peptides. (**E**) The bar graph showing a change in the frequency of the T cells after the stimulation. The activation of the T cells was determined with the upregulation of the CD25 surface marker.

SARS-CoV-2 specific IgG antibody analysis was performed in 1:128 and 1:256 titrations of serum isolated from blood. In SARS-CoV-2 antibody measurements, antibody development was observed in the vaccine groups, including the virus-administered group (Fig. 5A). According to the IgG ELISA result, the SARS-CoV-2 IgG antibody increase was significantly detected at 1:256 dilutions in the dose 10^14^ vaccinated group compared to the positive control group (non-vaccinated) (*p* = 0.001) (Fig. 5A). The neutralizing antibody study also showed a significant increase in both vaccine groups compared to the positive control at 1: 256, similar to the antibody levels (Fig. 5B). However, there was no significant change in gamma interferon responses from mouse spleen T cells without re-stimulation (Fig. 5C). Next, we wanted to determine the cytokine secretion profile and T cell frequencies between groups with a re-stimulation. Although it was not statistically significant, TNFα secretion was also seen to increase in the dose 10^14^ group (Fig. 5D). The increase of IL-2 in the dose 10^14^ vaccine group indicates that the mice vaccinated after the viral challenge show a Th1 type response (Fig. 5D). Also, when we compare SARS-CoV-2 infected non-vaccinated positive control with non-vaccinated and un-infected negative control, we determined that IL-10 cytokine, known as cytokine synthesis inhibitory factor, was significantly increased (Fig. 5D), suggesting downregulation of the expression of cytokines^22^. On the other hand, we wanted to determine a change in the proportion of spleen T cell subsets upon re-stimulation with SARS-CoV-2 peptides (Fig. 5E). Although total CD3+ T and CD4+ T cell populations did not increase in the vaccinated groups regarding control groups, CD25+ CD4+ T cell population was determined to increase in dose groups (Fig. 5E). Depending on the viral challenge, frequencies of CD3+ and CD4+ T lymphocytes significantly increased in the positive control group (non-vaccinated viral challenge group), while this increase was not observed in the vaccinated group (Fig. 5E). Only an increase in the amount of activated (CD25+) CD4+ T cells was observed in the vaccinated groups (Fig. 5E). This data showing that SARS-CoV-2 viral infection was caused to stimulate T cell response along with an increase of Th1 inhibitory Tr1 (T cell regulatory)-related IL-10 cytokine secretion and with the absence of Th2-related cytokine response. To sum up, the in vitro efficacy analysis of the challenge test showed that the presence of active T lymphocytes significantly increased in the dose 10^14^ vaccine group. The study indicated that viral dissemination was blocked by SARS-CoV-2 specific antibodies and neutralizing antibodies. It was also determined that the ADE effect was not observed, and also confirming that OZG-38.61.3 was non-replicative. As the cellular immune response, CD4+ T cell activation was present, especially at dose 10^14^, and T cell response was biased to the Th1 response type as desired in the immunization.

## Discussion

The SARS-CoV-2 virus caused one of the severest pandemics around the world. The safe and effective vaccine development for urgent use became more of an issue to end the global COVID-19 pandemic. There are widely approved various COVID-19 vaccines including Pfizer-BioNTech, Moderna, and other inactivated virus vaccines^1–4^. Here, we optimized an inactivated virus vaccine which includes the gamma irradiation process for the inactivation as an alternative to classical chemical inactivation methods so that there is no extra purification required. Previous studies showed that gamma-radiation can induce immunogenicity more effectively rather than conventional inactivation procedures^23^. Various chemical modifications such as formaldehyde or β-propiolactone are available in obtaining an effective and safe immunization in inactive vaccine production^24^. However, the chemical vaccine inactivation process is a time-consuming method due to the need for further purification process along with toxicity, chemical-dependent viral protein destruction, and product loss during final purification steps. The physical inactivation process in the three separate animal experiments in this and our previous study showed that intradermally administrations of gamma-irradiated SARS-CoV-2 vaccines were non-toxic and effective^19^. We have also determined the difference in loss of SARS-CoV-2 viral load upon gamma-irradiation and glutaraldehyde-based chemical inactivation process. We performed Single-radial-immunodiffusion (SRID) assay to determine functional Spike protein in the specimens by anti-SARS-CoV-2 Spike monoclonal antibody^25^, showing that chemically inactivated virus specimens lose viral load during the purification step due to the chemical ingredients (Supplementary Fig. 4). This suggests that gamma-irradiated inactivated vaccine preparation is a cost-effective approach.

We verified the inactivation degree of the virus post-gamma-irradiation treatment before immunization studies. The inactivation status of the vaccine candidates following the gamma-irradiation was determined by the 21-day culture of the vaccine on the Vero cells and MTT assay as previously reported in our first study on OZG-38.61.1^19^. Before immunization tests, we verified the OZG-38.61.3 candidates had included inactivated form of SARS-CoV-2. We applied the vaccine candidate (OZG-38.61.3) using the intradermal route in mice which decreased the requirement of a higher concentration of inactivated virus for proper immunization unlike most of the classical inactivated vaccine treatments^26,27^. There are plenty of Antigen-Presenting Cells in the dermis, thus studies reported that intradermal administration of inactivated vaccines reduced required antigenic doses around 20% or 30% of the standard amount of antigen used in the intramuscular (IM) or subcutaneous (SC) administration, which can induce immune responses equivalent to IM or SC administered inactivated vaccines with standard doses^28,29^. During the study, there had not been an International Unit (IU) analysis of SARS-CoV-2 particle number per volume. However, WHO has just reported vaccine dose determination standards as IU unit per ml^30^. Based on the standards, we revised the vaccine doses using IU units per ml (Supplementary Table 7).

It is much easier for the inactivated vaccine to reach the public, especially during pandemic periods. Because before the development of recombinant vaccines, the identification of the virus and its genome sequence must be revealed. However, inactivated vaccines can be used as vaccines after the virus is isolated, rapidly multiplied, and inactivated. However, the biggest problem of inactivated vaccine production is that vaccines can be developed rapidly, but it needs a longer time to reach large doses compared to recombinant vaccine production. Thus, inactivated vaccines can be developed more quickly, but they cannot be produced as quickly as recombinant vaccines. Different variations in SARS-CoV-2 strains may occur when producing large quantities (bulk) of the virus in a laboratory^31^. For this reason, 50% of the unit volume of virus isolates cultured in multi-layered flasks was frozen in each passage. While preparing the final product (OZG-38.61.3), frozen raw intermediate products were pooled. Thus, pre-pooling genomic characterization of individual variants between passages was made and the final product was created for a more effective and safer vaccine design. At the end of the vaccine production, the final product was found to contain most of the defined mutations in the SARS-CoV-2 strain. In addition, the SARS-CoV-2 virus was passaged 3 times for the isolation from the first donor and 6 times for the final production of OZG-38.61.3. The genome analysis of the OZG-38.61.3 vaccine in this study was found to retain > %99.5 homologies with the starting virus stock isolated from the COVID-19 patient. This may enable our inactive virus vaccine to be effective in a large population. Zeta-sizer along with Nanosight size analysis, proteome, and electron microscopic data showed that the OZG-38.61.3 vaccine preserved its compact structure despite gamma irradiation and lyophilization. However, we also detected aggregate formation, especially in electron microscope images. We added human serum albumin (< 0.02%) to the final product to increase the stability, prevent viral particles from adhering to the injection vial walls, and efficacy of the vaccine candidate^32^. Assessment of the residual Vero host cell protein and DNA level in each vaccine dose in this study showed that the protein level was < 4 ng and DNA was absent in the dose. This showed us that the vaccine production process is efficiently pure from the residual products.

In this study, we generated a prototype gamma-irradiated inactive SARS-CoV-2 vaccine (OZG-38.61.3) and assessed protective efficacy against the intranasal SARS-CoV-2 challenge in transgenic human ACE2 encoding mice. We demonstrate vaccine protection with substantial ~ 3 log10 reductions in mean viral loads in dose 10^14^ immunized mice compared with non-vaccinated infected positive control mice. We showed humoral and cellular immune responses against the SARS-CoV-2, including the neutralizing antibodies similar to those shown in BALB/c mice, without substantial toxicity. We have also determined subgroups of SARS-CoV-2 antibodies and showed that specifically IgG1 antibodies were upregulated along with total IgG (Supplementary Fig. 5).

When we performed the efficacy and safety test of the final product, OZG-38.61.3, vaccine candidate on BALB/ c mice at two different doses (10^13^ and 10^14^), the presence of SARS-CoV-2 specific neutralizing antibodies was significantly detected in the dose 10^14^ group. However, at both dose groups, significant IFNγ secretion from the spleen T cells was detected in comparison with the controls, illustrating that cellular immune response developed earlier than the humoral immune response. The fact that the neutralizing test was more accurate than the IgG ELISA analysis may be due to the increased levels of SARS-CoV-2 specific IgA and IgM antibodies^33–35^. Moreover, mice vaccinated with both doses showing a significant increase in T cell IFNγ responses and Th1 dominant cytokine release is additional evidence of vaccine efficacy. As no significant toxicity was encountered in the histopathological analysis of BALB/c mice vaccinated with both doses, a decision was taken to proceed to the challenge test.

A difficulty was faced with the intradermal vaccination of mice in that some of the study mice had skin injury due to the vaccination. This factor may have possibly reduced the efficacy of the intradermal vaccine tests and may be the reason behind the finding of a high standard deviation and inability to see a parallel neutralization capacity in each mouse. We used an ELISA-based microneutralization assay with MTT for assessing non-infected cell viability. The microneutralization ELISA assays have been used for the evaluation of neutralizing antibodies against the Influenza virus^25^. Because the sensitivity and specificity of the assay were shown closely related to the gold standard test results with plaque reduction neutralization test (PRNT), we wanted to quantify the neutralization capacity of the immunized mice serum by assessing MTT based colorimetric assay on ELISA. The recent report has also suggested using the microneutralization technique to assess neutralizing antibodies quantitatively in vitro^36^.

In the Challenge test, we collected oropharyngeal samples to determine the viral RNA copy number following the administration of the intranasal infective SARS-CoV-2 virus. In unvaccinated but virus-infected positive control mice viral RNA copy numbers at 48 and 96 h either did not change or were increased. However, in the groups of mice vaccinated with both doses, findings showed that copy numbers effectively decreased around 3 log10, and even a few mice had completely lost the viral load. X-rays were performed to search for a similar effect in the lung lobes, but the classic COVID-19 infection image was not observed in any of the groups. Also, qRT-PCR studies and histopathological lung analyses did not reveal pathological changes in the lung tissues. This may have been either due to the short 96-h infection period, or the low amount of virus (30,000 TCID50) used in the 96-h challenge test might not have been sufficient to descend into the lungs during this period. There are also previous reports regarding the absence of viral load in the lung tissue being due to the amount of infected dose or the virus could be detected in the specific locations of the lung^1,37^. When neutralizing antibody capacity of mice vaccinated in the Challenge test was analyzed, both doses of vaccination were observed to significantly neutralize the SARS-CoV-2. This is following the previous finding of a reduction in viral RNA copy numbers in immunized mice.

When we looked at the neutralizing antibody capacity of mice vaccinated within the scope of the Challenge test, we observed that both doses of vaccination could significantly neutralize SARS-CoV-2. This shows us that the reduction in viral RNA copy rates is consistent. However, when we looked at the T cell response, we could not see any difference in IFNγ release. Presumably, because groups of mice are infected with the virus, T cells may already be stimulated and this may not make a difference in IFNγ release. A significant decrease in CD3+ and CD4+ T cell ratios and an increase in CD25+ CD4+ T cell ratio show that these cells have already been activated. On the other hand, the fact that the virus was neutralized here prevented the increase in CD3+ T cell proportion, therefore viral challenge resulted in only the increase of active T cells. When we looked at spleen T cells that were not re-stimulated, we detected Th1-type cytokine release, as we expected, especially in the 10^14^ dose vaccine group. On the other hand, the significant increase in the ratios of total CD3+ and CD4+ T cells and the ratios of activated (CD25+) CD8+ T cells and the level of the Th1 cytokine and inhibitor IL-10 between the negative control and positive control mice that had an only viral infection. It shows that in a short time such as 96 h, it started to generate T cell response more effectively than antibody response. This has shown that T cell response occurs in individuals exposed to the virus without sufficient time for neutralizing antibody formation.

The OZG-38.61.3 vaccine candidate is an inactivated vaccine form. We have shown in our previous article that OZG-38.61.3 has two advantages over other conventional inactivated vaccines. WHO guideline about the development of vaccines with intradermal delivery in low- and middle-income countries suggested that adjuvants might need to be reduced or even removed (Julian Hickling and Rebecca Jones, 2019). Secondly, when compared to the glutaraldehyde inactivated sample of SARS-CoV-2 sample inactivated by gamma irradiation, the virus particle count and Spike protein structure are preserved in the final product; In the glutaraldehyde inactivated sample, the loss was observed due to purification processes. AstraZeneca, Pfizer-BioNTech and Moderna COVID-19 mRNA vaccines are new-generation vaccine forms and the long-term side-effect profile of the vaccine has not yet been determined. However, the classic vaccine including an inactivated virus has been used for long years^38^. Therefore, OZG-38.61.3 is a classical vaccine form despite gamma irradiation and intradermal applications. OZG-38.61.3 was developed with virus strains containing highly frequent mutations including D614G^39,40^. Furthermore, to determine the neutralization capacity of the OZG-38.61.3 vaccine against the new SARS-CoV-2 strain collected serum specimens from immunized rodents were incubated with 1000 TCID50 doses of SARS-CoV-2 Brazilian strain (Supplementary Fig. 3). The study suggested that the OZG-38.61.3 vaccine candidate has a sufficient neutralization efficacy against a new SARS-CoV-2 strain. In the neutralization tests performed with the Brazilian strain, it has been shown that the vaccine maintains its effectiveness and has a similar neutralization capacity (Supplementary Fig. 3).

In summary, this study demonstrated that the OZG-38.61.3 vaccine candidates created with gamma-irradiated inactivated SARS-CoV-2 viruses produced neutralizing antibodies, especially effective in the 10^14^ viral RNA copy formulation, and this was effective in protecting transgenic human ACE2 expressing mice against the SARS-CoV-2 virus. Vaccine candidates were demonstrated to be safe to the tissues of BALB/c and transgenic mice. This study will lead to the initiation of Phase 1 clinical application of the vaccine for the COVID-19 pandemic.

## Supplementary Information

Supplementary Information.
