# Supplementary material for: Gamma-irradiated SARS-CoV-2 vaccine candidate, OZG-38.61.3, confers protection from SARS-CoV-2 challenge in human ACEII-transgenic mice
Source: Sci Rep. 2021 Aug 4;11:15799. doi: 10.1038/s41598-021-95086-4 (PMC8339061; doi:10.1038/s41598-021-95086-4)
Supplement: Supplementary file 1 — Supplementary Information. [file 41598_2021_95086_MOESM1_ESM.pdf]

Supplementary Materials

Gamma-irradiated SARS-CoV-2 vaccine candidate, OZG-38.61.3, confers protection from SARS-CoV-2 challenge in human ACEII-transgenic mice

Raife Dilek Turan, Cihan Tastan, Derya Dilek Kancagi, Bulut Yurtsever, Gozde Sir Karakus, Samed Ozer, Selen Abanuz, Didem Cakirsoy, Gamze Tumentemur, Sevda Demir, Utku Seyis, Recai Kuzay, Muhammer Elek, Miyase Ezgi Kocaoglu, Gurcan Ertop, Serap Arbak, Merve Acikel Elmas, Cansu Hemsinlioglu, Ozden Hatirnaz Ng, Sezer Akyoney, Ilayda Sahin, Cavit Kerem Kayhan, Fatma Tokat, Gurler Akpinar, Murat Kasap, Ayse Sesin Kocagoz, Ugur Ozbek, Dilek Telci, Fikrettin Sahin, Koray Yalcin, Siret Ratip, Umit Ince, Ercument Ovali

Supplementary figures/tables

Study groups	Earring No	Day 0	Day 5	Day 9	Day 14	Day 21	Day 26/1	Day 26/2	Day 27/1	Day 27/2	Day 28/1	Day 28/2	Day 29
Negative control	**40**	29,00	29,30	25,60	32,10	33,00	NA	NA	NA	NA	NA	NA	33,90
	**41**	24,20	24,70	31,20	26,20	27,00	NA	NA	NA	NA	NA	NA	27,30
	**42**	19,00	20,20	21,10	21,30	22,40	NA	NA	NA	NA	NA	NA	21,90
	**43**	22,00	23,00	23,10	23,10	24,10	NA	NA	NA	NA	NA	NA	24,60
	**44**	20,10	21,10	22,00	22,20	23,30	NA	NA	NA	NA	NA	NA	24,00
	**Average**	22,86	23,66	24,60	24,98	25,96	NA	NA	NA	NA	NA	NA	26,34
	**Standard deviation**	3,96	3,60	4,06	4,39	4,30	NA	NA	NA	NA	NA	NA	4,64
Pozitive Control	**45**	21,30	22,00	23,10	23,00	24,20	24,60	24,10	24,30	24,00	23,10	22,70	23,70
	**46**	17,80	17,60	18,90	19,30	19,90	19,50	19,50	19,40	20,40	19,90	19,60	19,00
	**47**	23,80	24,50	26,10	26,70	27,00	27,40	26,90	27,10	26,80	27,40	26,70	26,70
	**48**	25,20	26,20	27,50	27,30	28,40	29,10	28,10	28,90	28,40	28,40	28,00	28,50
	**49**	26,60	28,00	29,00	30,30	30,80	31,00	30,50	31,00	30,80	30,50	30,60	31,20
	**50**	22,20	23,00	24,00	25,00	25,00	25,20	25,10	25,60	26,10	25,40	25,20	25,50
	**Average**	22,82	23,55	24,77	25,27	25,88	26,13	25,70	26,05	26,08	25,78	25,47	25,77
	**Standard deviation**	3,12	3,63	3,60	3,80	3,77	4,03	3,78	4,03	3,60	3,84	3,91	4,20
Dose 1 x	**51**	28,80	29,00	30,60	30,50	31,30	31,80	31,20	31,50	31,00	31,20	25,30	32,00

	**52**	22,10	22,70	23,90	24,90	24,70	25,00	25,10	25,10	25,70	25,40	30,80	25,70
	**53**	27,80	28,50	30,10	30,30	30,80	32,10	31,20	31,50	31,20	30,80	31,00	31,70
	**54**	26,40	27,80	30,60	30,20	30,60	31,30	31,40	31,40	31,20	31,50	30,90	31,30
	**55**	25,20	26,10	27,30	27,30	27,60	28,60	28,20	28,70	29,10	28,20	27,60	28,40
	**56**	25,10	25,70	27,70	27,60	27,80	28,90	29,00	29,20	29,00	29,30	28,70	29,70
	**57**	30,10	31,20	33,20	33,30	34,00	35,00	31,00	35,00	33,80	34,80	34,10	34,30
	**Average**	26,50	27,29	29,06	29,16	29,54	30,39	29,59	30,34	30,14	30,17	29,77	30,44
	**Standard deviation**	2,68	2,74	3,01	2,75	3,05	3,20	2,34	3,08	2,53	2,95	2,84	2,79
**Dose 1 x 10^{14}**	**58**	23,20	24,10	24,50	25,40	26,00	26,10	25,80	26,70	26,10	26,20	26,20	26,00
	**59**	27,90	28,80	30,10	31,20	31,90	32,50	31,60	32,50	32,20	31,80	31,70	32,40
	**60**	20,50	21,20	21,70	22,70	23,50	23,70	23,20	23,50	22,80	23,40	22,90	23,30
	**61**	28,50	28,90	30,20	31,30	31,80	32,00	32,70	32,10	32,30	31,90	32,00	32,60
	**62**	24,60	24,80	26,20	26,40	27,10	27,70	27,60	28,00	27,30	27,50	27,90	27,30
	**63**	26,10	26,70	28,40	28,60	29,50	29,50	29,50	29,70	29,80	29,50	29,30	30,20
	**64**	25,30	26,10	27,90	28,30	29,20	30,00	29,90	30,12	29,70	29,60	29,40	21,70
	**Average**	25,16	25,80	27,00	27,70	28,43	28,79	28,61	28,95	28,60	28,56	28,49	27,64
	**Standard deviation**	2,75	2,73	3,10	3,12	3,09	3,17	3,32	3,17	3,44	3,08	3,19	4,30

Supplementary Table 1: Viral Challenge Individual Weight (gr) Changes Evaluation Chart.

**Nucleotide Position**	**Gene/ Region**	**Gene Product**	**Nucleotide Exchange (Ref/Alt)**	**Amino acid exchange**	**Mutation Type**
**241**	5' UTR	NA	C/T	NA	Non-coding
**913**	ORF1ab	Nsp2	C/T	S36S	Synonymous
**3037**	OF1ab	Nsp3	C/T	F106F	Synonymous
**12809**	OF1ab	Nsp9	C/T	L42F	Missense
**13170**	ORF1ab	Nsp10	C/T	T49I	Missense
**14408**	ORF1ab	RNA-dependent RNA Polymerase	C/T	P323L	Missense
**14676**	ORF1ab	RNA-dependent RNA Polymerase	C/T	P412P	Synonymous
**21648**	S	Spike glycoprotein	C/T	T29I	Missense
**21789**	S	Spike glycoprotein	C/T	T76I	Missense
**21846**	S	Spike glycoprotein	C/T	T95I	Missense
**22036**	S	Spike glycoprotein	A/C	R158S	Missense
**22117**	S	Spike glycoprotein	T/G	N185K	Missense
**23014**	S	Spike glycoprotein	A/T	E484D	Missense
**23403**	S	Spike glycoprotein	A/G	D614G	Missense
**23520**	S	Spike glycoprotein	C/T	E653D	Missense
**23997**	S	Spike glycoprotein	C/T	P812L	Missense
**24538**	S	Spike glycoprotein	A/C	Q992H	Missense
**26324**	E	Envelope protein	T/C	L27S	Missense
**26542**	M	Membrane glycoprotein	C/T	T7I	Missense
**26559**	M	Membrane glycoprotein	C/T	L13F	Missense
**28881**	N	Nucleocapsid phosphoprotein	G/A	R204K	Missense
**28882**	N	Nucleocapsid phosphoprotein	G/A	R204K	Missense
**28883**	N	Nucleocapsid phosphoprotein	G/C	G204R	Missense

Supplementary Table 2: Viral Genome Sequencing analysis of fractionated SARS-CoV-2 strains forming OZG-38.61.3 vaccine candidate.

Parameter	Method	Limit	Result
**Appearance**	Visual inspection	Dry Grayish Powder	Passed
**Identity**	Single Radial Immunodiffusion (SRID)	Positive	Positive
	TEM	Positive	Positive
**Size And Particle Analysis**	Nanosight<br>Zetasizer	Size <200 Nm<br>Particle >$1x10^8$ Ml	Passed
**Virus Copy Number/Dose Analysis**	qRT-PCR	*≥ $1x10^{13}$<br>*≥ $1x10^{14}$	Passed
**Biochemical Analysis**	Ph, Na, Cl, K, Ca, Glucose, Total Protein, Albumin	Reference Interval<br>≥ 70 µg total protein/ dose	Passed
**Relative Humidity**	Moisture Analyzer	<%3	Passed
**Purity**	Vero Protein ELISA	<4 ng	Passed
	Vero Dna Nanodrop	<10 ng	Passed
**Replicative Virus Test**	Real-Time Cell Analysis (RTCA) or MTT	-	Passed
**Proteome**	Mass Spectrometer	-	Passed
**Microbiological Quality Control**	Culture	Negative	Negative
	Gram Stain	Negative	Negative
**Fungi**	Culture	Negative	Negative
**Mycoplasma**	PCR	Negative	Negative
**Endotoxin**	Lal Test	<0.5 IU/ml	Passed
**Stability/ Efficiency**	Single Radial Immunodiffusion (SRID)	Positive	Efficiency analysis of

			0,1,6,12,24 and 26 months in 4-24˚C storage conditions.

Supplementary Table 3: Quality Control tests of the lyophilized product of OZG-38.61.3 vaccine candidate. * Infective SARS-CoV-2 viral copy number in a dose.

**Accession**	**Description**	**Coverage [%]**	# PSM	**# Peptide**	**# AAs**	**MW [kDa]**	**calc. pI**	**Score Sequest HT**
**A0A6G7SLW8**	Orf1ab polyprotein OS=Severe acute respiratory syndrome coronavirus 2	1,02860	19	4	7097	793,7	6,76	0
**A0A6C0T6Z7**	Nucleoprotein OS=Severe acute respiratory syndrome coronavirus 2 O	42,0047	30	12	419	45,6	10,07	56,69
**A0A679G9E9**	S protein OS=Severe acute respiratory syndrome coronavirus 2	0,54988	1	1	1273	141,1	6,65	0
**A0A6C0N6C6**	Nonstructural protein NS8 OS=Severe acute respiratory syndrome coronavirus2	23,9669	2	2	121	13,8	5,73	0
**A0A6H2LA46**	ORF1ab polyprotein OS=Severe acute respiratory syndrome coronavirus 2	1,02874	19	4	7096	792,5	6,7	0
**A0A6H2LAX8**	ORF1ab polyprotein OS=Severe acute respiratory syndrome coronavirus 2	1,02874	19	4	7096	793,5	6,76	0
**A0A6H1PMM5**	Surface glycoprotein OS=Severe acute respiratory syndrome coronavirus 2	0,54988	1	1	1273	141,1	6,61	0
**A0A6H2L905**	ORF1ab polyprotein OS=Severe acute respiratory syndrome coronavirus 2	1,02874	19	4	7096	793,5	6,76	0
**A0A6H2LC46**	Surface glycoprotein OS=Severe acute respiratory syndrome coronavirus 2	0,54988	1	1	1273	140,3	6,76	0
**A0A6H2LCV0**	ORF1ab polyprotein OS=Severe acute respiratory syndrome coronavirus 2	1,02874	19	4	7096	793,5	6,76	0
**A0A6H2L673**	Surface glycoprotein OS=Severe acute respiratory syndrome coronavirus 2	0,54988	1	1	1273	141,1	6,73	0
**A0A6H2L7S6**	ORF1ab polyprotein OS=Severe acute respiratory syndrome coronavirus 2	1,02874	19	4	7096	793,7	6,76	0

**A0A6H2L8K8**	ORF1ab polyprotein OS=Severe acute respiratory syndrome coronavirus 2	1,02874	19	4	7096	793,5	6,62	0
**A0A6H2L9B8**	ORF1ab polyprotein OS=Severe acute respiratory syndrome coronavirus 2	1,02874	19	4	7096	793,6	6,76	0
**A0A6H2LBL6**	ORF1ab polyprotein OS=Severe acute respiratory syndrome coronavirus 2	1,02874	19	4	7096	793,6	6,76	0
**A0A6H2LDA7**	ORF1ab polyprotein OS=Severe acute respiratory syndrome coronavirus 2	1,02874	19	4	7096	792,9	6,64	0
**A0A6H2LBC7**	ORF1ab polyprotein OS=Severe acute respiratory syndrome coronavirus 2	1,02874	19	4	7096	793,2	6,68	0
**A0A6H2LA93**	Nucleocapsid phosphoprotein OS=Severe acute respiratory syndrome coronavirus 2	42,0047	30	12	419	45,6	10,07	56,69
**A0A6H2L973**	ORF1ab polyprotein OS=Severe acute respiratory syndrome coronavirus 2	1,02874	19	4	7096	793	6,81	0
**A0A6H2L8H9**	ORF1ab polyprotein OS=Severe acute respiratory syndrome coronavirus 2	1,02874	19	4	7096	793,6	6,76	0
**A0A6H2LAC5**	ORF1ab polyprotein OS=Severe acute respiratory syndrome coronavirus 2	1,02874	19	4	7096	793,3	6,71	0
**A0A6H2LBD2**	Surface glycoprotein OS=Severe acute respiratory syndrome coronavirus 2	0,54988	1	1	1273	141	6,73	0
**A0A6H2L6R1**	ORF1ab polyprotein (Fragment) OS=Severe acute respiratory syndrome coronavirus 2	1,04539	19	4	6983	780,6	6,77	0
**A0A6H2L7I9**	ORF1ab polyprotein OS=Severe acute respiratory syndrome coronavirus 2	1,02874	19	4	7096	793,2	6,7	0
**A0A6H2LC40**	Surface glycoprotein OS=Severe acute respiratory syndrome coronavirus 2	0,54988	1	1	1273	140,9	6,73	0
**A0A6H2LBU1**	Surface glycoprotein (Fragment) OS=Severe acute respiratory syndrome coronavirus 2	0,61946	1	1	1130	124,9	6,44	0
**A0A6H2LC49**	ORF1ab polyprotein OS=Severe acute respiratory syndrome coronavirus 2	1,02874	19	4	7096	793,6	6,76	0
**A0A6H2L683**	Surface glycoprotein OS=Severe acute respiratory syndrome coronavirus 2	0,54988	1	1	1273	141,1	6,81	0
**A0A6H2LDI3**	ORF1ab polyprotein OS=Severe acute respiratory syndrome coronavirus 2	1,02874	19	4	7096	793,5	6,73	0
**A0A6H2L877**	ORF1ab polyprotein OS=Severe acute respiratory syndrome coronavirus 2	1,02874	19	4	7096	793,6	6,76	0
**A0A6H2L915**	Nucleocapsid phosphoprotein OS=Severe acute respiratory syndrome coronavirus 2	42,0047	30	12	419	45,7	10,05	56,69

**A0A6H2LBN3**	ORF8 protein OS=Severe acute respiratory syndrome coronavirus 2	23,9669	2	2	121	13,8	5,73	0
**A0A6H2L880**	ORF1ab polyprotein OS=Severe acute respiratory syndrome coronavirus 2	1,02874	19	4	7096	793,1	6,62	0
**A0A6H2LAL1**	ORF1ab polyprotein OS=Severe acute respiratory syndrome coronavirus 2	1,02874	19	4	7096	793,6	6,76	0
**A0A6H2L964**	Surface glycoprotein OS=Severe acute respiratory syndrome coronavirus 2	0,54988	1	1	1273	141,1	6,73	0
**A0A6H2L9W1**	ORF1ab polyprotein OS=Severe acute respiratory syndrome coronavirus 2	1,02874	19	4	7096	793,6	6,76	0
**A0A6H2LCH0**	ORF1ab polyprotein OS=Severe acute respiratory syndrome coronavirus 2	1,02874	19	4	7096	793,6	6,76	0
**A0A6H2L702**	Surface glycoprotein OS=Severe acute respiratory syndrome coronavirus 2	0,54988	1	1	1273	141,1	6,65	0
**A0A6H1PRW5**	ORF1ab polyprotein OS=Severe acute respiratory syndrome coronavirus 2	1,02874	19	4	7096	793,6	6,76	0
**A0A6H2L7Z5**	Nucleocapsid phosphoprotein OS=Severe acute respiratory syndrome coronavirus 2	42,0047	30	12	419	45,6	10,07	56,69
**A0A6H2L6T0**	Nucleocapsid phosphoprotein OS=Severe acute respiratory syndrome coronavirus 2	42,0047	30	12	419	45,6	10,07	56,69
**A0A6H2LA09**	ORF1ab polyprotein OS=Severe acute respiratory syndrome coronavirus 2	1,02874	19	4	7096	793	6,71	0
**A0A6H2LCI8**	ORF1ab polyprotein OS=Severe acute respiratory syndrome coronavirus 2	1,02874	19	4	7096	793,6	6,76	0
**A0A6H2L7Z4**	ORF1ab polyprotein OS=Severe acute respiratory syndrome coronavirus 2	1,02874	19	4	7096	793,7	6,76	0
**A0A6H2LA55**	ORF1ab polyprotein OS=Severe acute respiratory syndrome coronavirus 2	1,02874	19	4	7096	793,6	6,8	0
**A0A6H2L7V1**	Nucleocapsid phosphoprotein OS=Severe acute respiratory syndrome coronavirus 2	42,0047	30	12	419	45,6	10,07	56,69
**A0A6H2LD79**	ORF1ab polyprotein OS=Severe acute respiratory syndrome coronavirus 2	1,02874	19	4	7096	793,6	6,76	0
**A0A6H2L9L2**	ORF1ab polyprotein OS=Severe acute respiratory syndrome coronavirus 2	1,02874	19	4	7096	793,2	6,71	0
**A0A6H2L6I9**	ORF1ab polyprotein OS=Severe acute respiratory syndrome coronavirus 2	1,02874	19	4	7096	792,9	6,7	0
**A0A6H2LAG5**	Surface glycoprotein OS=Severe acute respiratory syndrome coronavirus 2	0,54988	1	1	1273	140,6	6,04	0
**A0A6H2L8N1**	ORF1ab polyprotein OS=Severe acute respiratory syndrome coronavirus 2	1,02874	19	4	7096	793,6	6,76	0

**A0A6G7SLV1**	Orf1ab polyprotein OS=Severe acute respiratory syndrome coronavirus 2	1,02874	19	4	7096	793,6	6,76	0
**A0A6H2EH11**	ORF1ab polyprotein OS=Severe acute respiratory syndrome coronavirus 2	1,02874	19	4	7096	793,5	6,76	0
**A0A6H0QT63**	ORF1ab polyprotein OS=Severe acute respiratory syndrome coronavirus 2	1,02874	19	4	7096	793,5	6,76	0
**A0A6H2L7L3**	Surface glycoprotein OS=Severe acute respiratory syndrome coronavirus 2	0,54988	1	1	1273	141	6,81	0
**A0A6H2L5M8**	Nucleocapsid phosphoprotein OS=Severe acute respiratory syndrome coronavirus 2	42,0047	30	12	419	45,7	10,08	56,69
**A0A6H2LAI7**	ORF1ab polyprotein OS=Severe acute respiratory syndrome coronavirus 2	1,02874	19	4	7096	793,6	6,76	0
**A0A6H2LEE1**	ORF1ab polyprotein OS=Severe acute respiratory syndrome coronavirus 2	1,02874	19	4	7096	793,6	6,76	0
**A0A6H2LBP4**	ORF1ab polyprotein OS=Severe acute respiratory syndrome coronavirus 2	1,02874	19	4	7096	793,6	6,74	0
**A0A6H2L794**	ORF1ab polyprotein OS=Severe acute respiratory syndrome coronavirus 2	1,02874	19	4	7096	793,6	6,76	0
**A0A6H2LCV8**	ORF1ab polyprotein OS=Severe acute respiratory syndrome coronavirus 2	1,02874	19	4	7096	793,6	6,76	0
**A0A6C0RS15**	2'-O-methyltransferase OS=Severe acute respiratory syndrome coronavirus2	1,028749	19	4	7096	793,6	6,76	0
**A0A6G7SLW0**	Orf1ab polyprotein OS=Severe acute respiratory syndrome coronavirus 2	1,02874	19	4	7096	793,5	6,74	0
**A0A6H2LB79**	ORF1ab polyprotein OS=Severe acute respiratory syndrome coronavirus 2	1,028749	19	4	7096	793,6	6,76	0
**A0A6H2LB01**	ORF1ab polyprotein OS=Severe acute respiratory syndrome coronavirus 2	1,02874	19	4	7096	792,8	6,71	0

Supplementary Table 4: LC-MS/MS results of candidate vaccine sample developed against the SARS-CoV-2 virus. OX=2697049.

Table 5a:

	Control	$1x10^{13}$	$1x10^{14}$
**Total Body Weight**	35,16±0,9	35,16±0,8	35,16±0,7
**Lung**	1,34±0,01	1,34±0,03	1,343±0,02
**Liver**	2,59±0,14	2,59±0,19	2,59±0,18
**Spleen**	1,22±0,01	1,22±0,01	1,22±0,01
**Kidney**	2,64±0,03	2,64±0,04	2,64±0,05

Table 5b:

	Control	$1x10^{13}$	$1x10^{14}$
**Inflammation**	2/5*	1/5*	1/5*
**Hemorrhage**	0/5	0/5	0/5
**Eosinophil**	0/5	1/5	0/5

Table 5c:

	Control	$1x10^{13}$	$1x10^{14}$
**Inflammation**	0/5	2/5*	0/5
**Hemorrhage**	0/5	0/5	0/5
**Eosinophil**	0/5	0/5	0/5

Table 5d:

	Control	$1x10^{13}$	$1x10^{14}$
**Inflammation**	1/5*	1/5*	0/5
**Hemorrhage**	0/5	0/5	0/5
**Eosinophil**	0/5	0/5	0/5

Supplementary Table 5: Organ weights and histopathological analysis vaccinated Balb/c mice. A. Organ Weights Chart**.** Histological Analysis of **B.** the lung. **C.** the kidney **D.** the liver tissues. Control mice, n=5, Dose 10^{13} vaccinated mice, n=5, and Dose 10^{14} vaccinated mice, n=5. *****mild inflammation

Groups	Earring no	Lung H-Score
Negative Control (Human lung and placental tissue)	**40**	0,00
	**41**	0,00
	**42**	1,00
	**43**	2,00
	**44**	0,00
	**Average**	0,60
	**Standard deviation**	0,89
Positive Control (SARS-CoV-2 infected mice)	**45**	46,00
	**46**	76,00
	**47**	59,00
	**48**	34,00
	**49**	71,00
	**50**	95,00
	**Average**	63,50
Dose 1×10^{13} administrated mice	**Standard deviation**	21,92
	**51**	26,00
	**52**	42,00
	**53**	38,00
	**54**	29,00
	**55**	28,00

	**56**	33,00
	**57**	37,00
	**Ortalama**	33,29
	**Standard deviation**	5,94
**Dose 1 x 10 ^{14} administrated mice**	**58**	0,00
	**59**	1,00
	**60**	3,00
	**61**	2,00
	**62**	0,00
	**63**	0,00
	**64**	0,00
	**Average**	0,86
	**Standard deviation**	1,21
**Overall**	**Average**	24,92
	**Standard deviation**	28,18

Supplementary Table 6. Lung Tissue Immunohistochemistry Image (H-score Analysis) Individual Evaluation Chart. In the statistical analysis of these analyzes, the control groups and dose groups were compared using the Student's t-test method. Significance between groups was added to the graph. ($p < 0.05$).

	Cq	Formül	Anti-Log	x 5x10^3 copy/ml dilution factor	x 5x10^4 copy/ml dilution factor	x 5x10^5 copy/ml dilution factor	x 5x10^6 copy/ml dilution factor
1st test							
SC2 10^0	18,79	6,69	4920796,42				
SC2 10^1	21,14	5,65	448964,90	$2{,}24x10^{9}$			
SC2 10^2	24,33	4,24	17406,36		$8{,}7x10^{8}$		
SC2 10^3	27,48	2,85	702,91			$35x10^{7}$	
2nd test							
SC2 10^2	24,85	4,01	10247,54		$5{,}12x10^{8}$		
SC2 10^3	28,38	2,45	280,98			$14x10^{7}$	
SC2 10^4	31,33	1,14	13,91				$6{,}95x10^{7}$

Supplementary Table 7. Calculation of copy number based on Cq value based on World Health Organization (WHO) SARS-CoV-2 RNA copy number standard analysis (International Unit per ml; IU/ml).

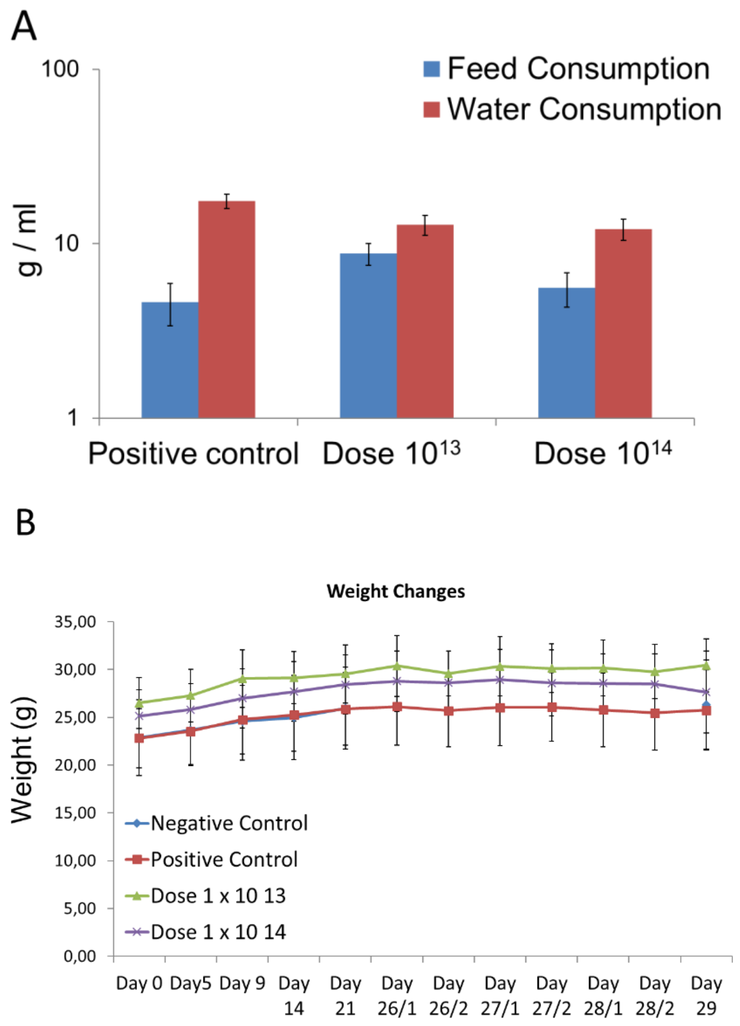

Supplementary Figure 1. Health state of challenge test mice. A. Feed and Water consumption per day post-infection of the positive control group, dose $1x10^{13,}$ and $1x10^{14}$ groups. **B.** Monitoring**.** Bodyweight changes during challenge experiments.

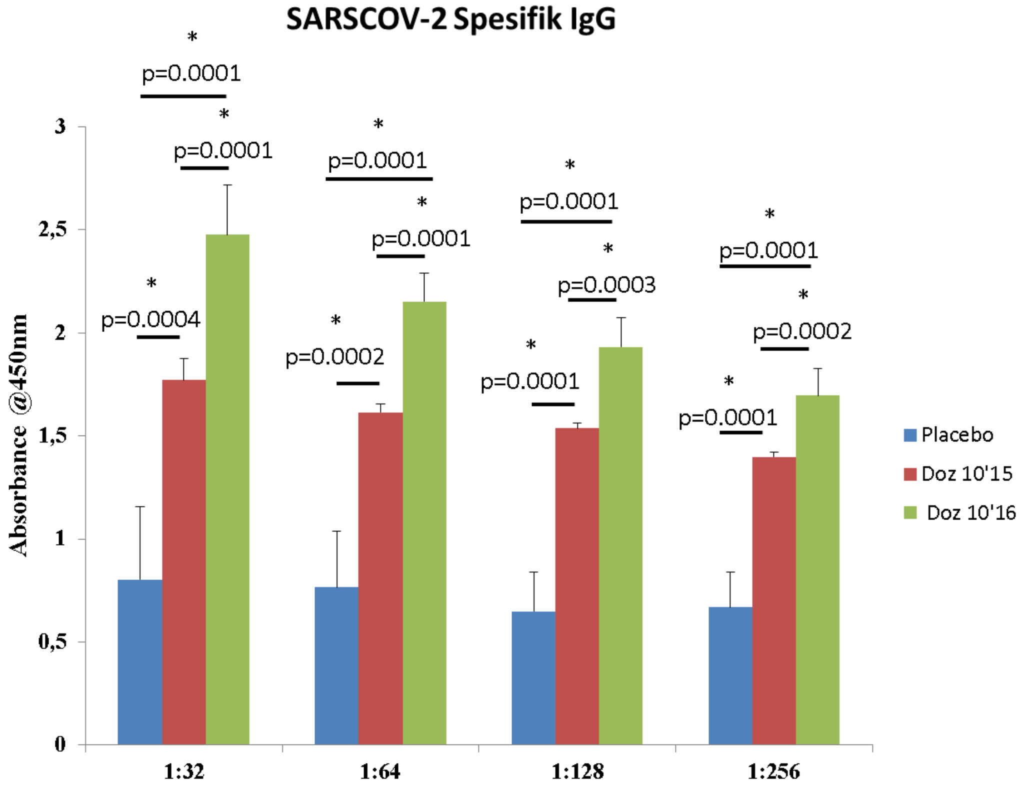

Supplementary Figure 2. Repeat Dose Study in Rodents Immune Responses (SARS-CoV-2 Specific IgG Analysis). The 21-day administrated rodent serums were studied with the Creative Diagnostic SARS-CoV-2 Specific IgG Elisa kit by diluting in 1:32, 1:64, 1:128, 1:256 dilution factors of test groups including placebo, dose $1x10^{15}$, dose $1x10^{16}$ vaccine. Measured absorbance at 450nm, absorbance averages, and absorbance standard deviation values are indicated.

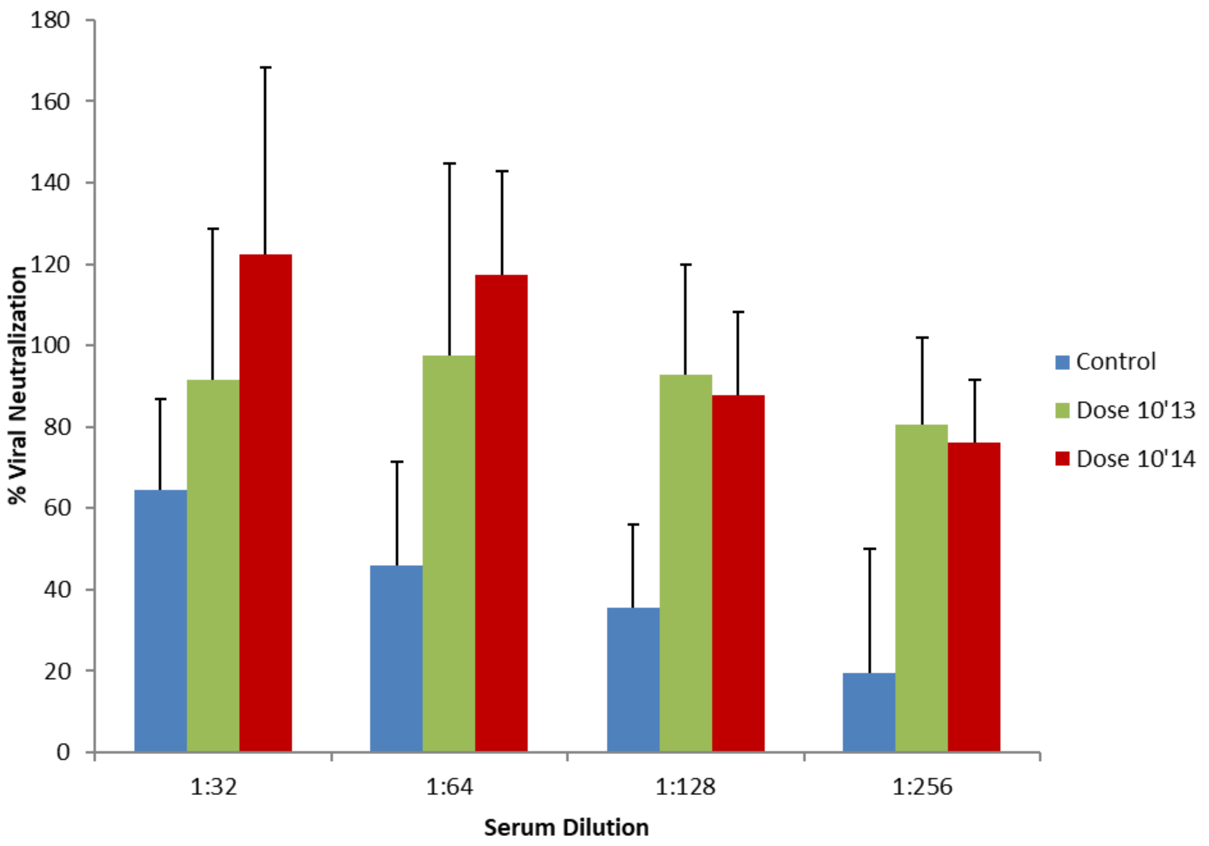

Supplementary Figure 3. Neutralization analysis in repeated dose administrated rodents. In a repeated-dose study in rodents, rats were treated with placebo, dose 10^{13}, and dose 10^{14} vaccine were diluted in 1:32, 1:64, 1:128, 1:256 dilution factors and cultured on Vero cells after treatment with 1000 TCID50 Brazilian strain of SARS-CoV-2 virus. 96 hours later, neutralization efficacy was determined by MTT analysis.

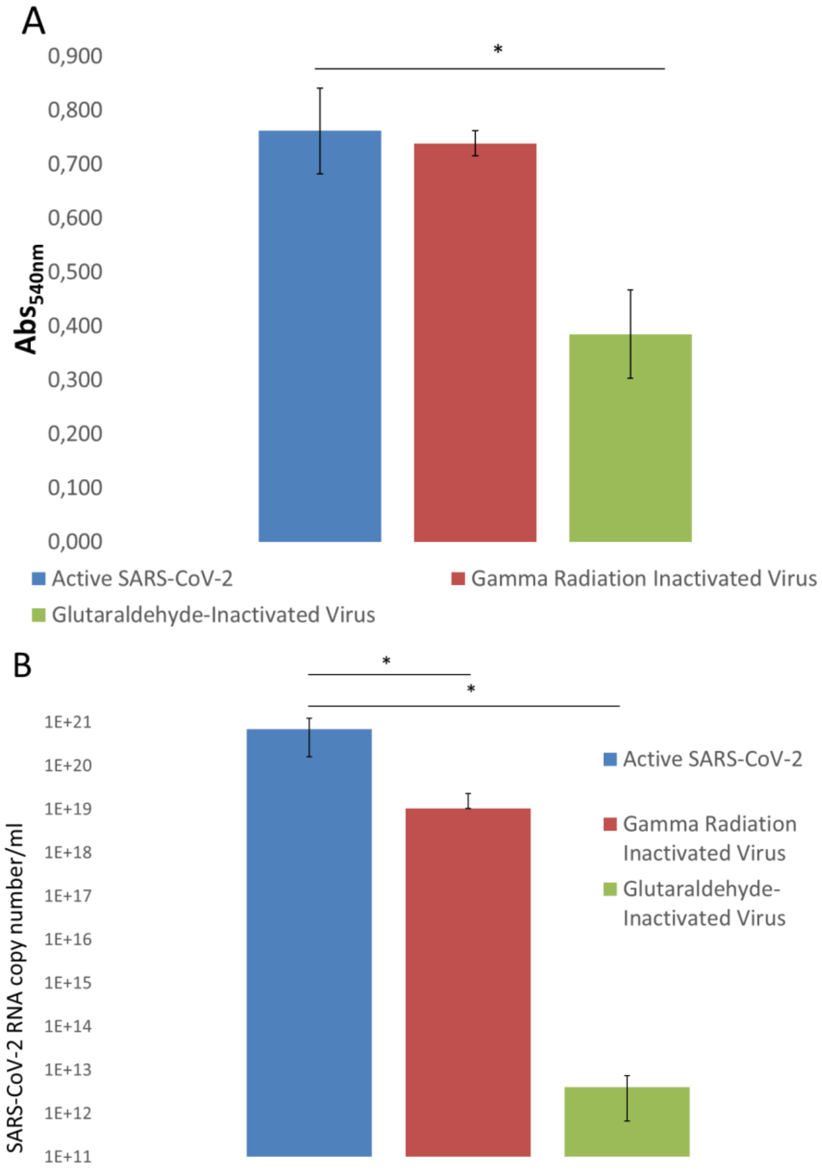

Supplementary Figure 4. The difference in loss of Viral load upon Gamma-irradiation and the Glutaraldehyde-based chemical inactivation process. An equal dose of SARS-CoV-2 virus was inactivated either with 2.5% glutaraldehyde in PBS (0.1 M, pH 7.2) for 2.5 h and isolated through 100 kDa Amicon filter or 25 kGy gamma-irradiation. Next, Single-radial-immunodiffusion (SRID) assay was performed to determine functional Spike protein in the

specimens by anti-SARS-CoV-2 Spike monoclonal antibody (Elabscience, E-AB-V1002). **A.** Spike protein-monoclonal antibody interaction was assessed by microplate reader at 540nm absorbance. **B.** SARS-CoV-2 RNA copy number of the specimens which were untreated control, gamma-irradiated, or chemically inactivated determined by RT-PCR. Experiments were repeated three times. *p<0.05

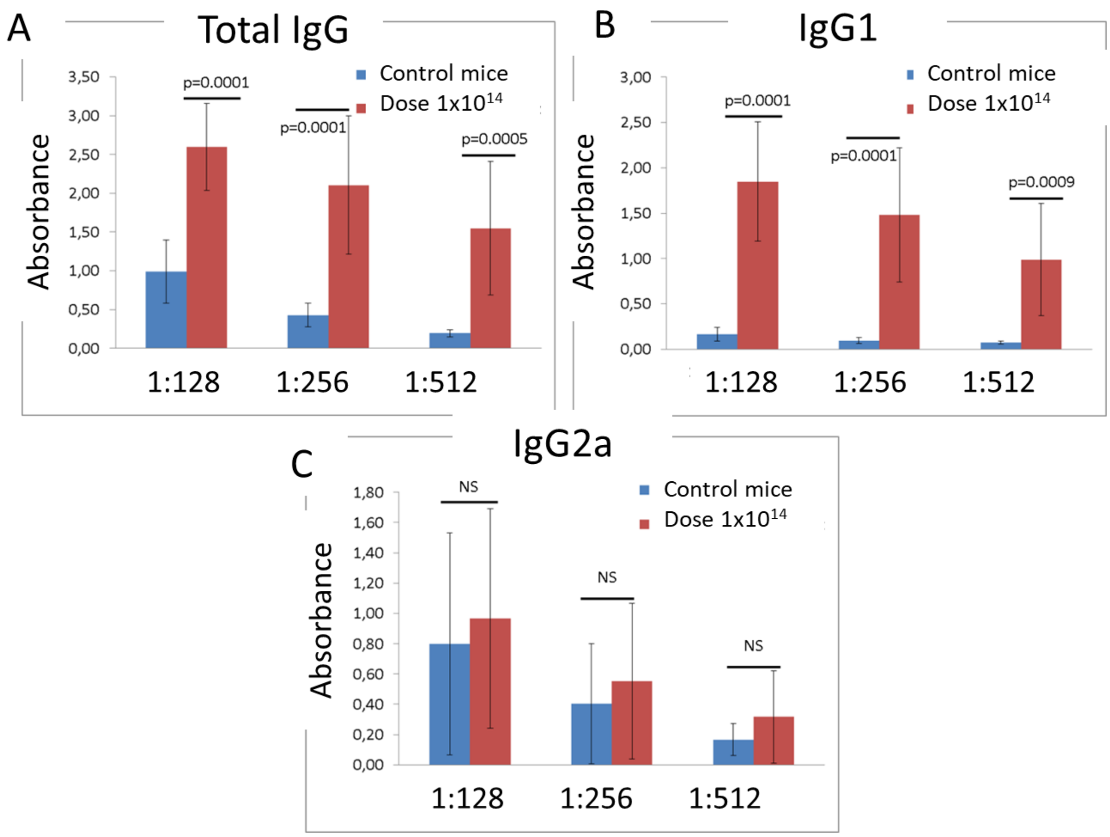

Supplementary Figure 5. Subgroups of SARS-CoV-2 antibody produced upon immunization of BALB/c mice. A. Total IgG. B. IgG1 and C. IgG2a
